# Development of the Reporting Infographics and Visual Abstracts of Comparative studies (RIVA-C) checklist and guide

**DOI:** 10.1136/bmjebm-2023-112784

**Published:** 2024-01-19

**Authors:** Joshua R Zadro, Giovanni E Ferreira, Will Stahl-Timmins, Veronika Egger, Mark R Elkins, Andrew R Gamble, Mary O'Keeffe, Kirsten J McCaffery, Ian A Harris, Clare L Ardern, Courtney A West, Chris G Maher, Tammy C Hoffmann

**Affiliations:** 1 Sydney Musculoskeletal Health, Institute for Musculoskeletal Health, Sydney School of Public Health, Faculty of Medicine and Health, The University of Sydney and Sydney Local Health District, Sydney, NSW, Australia; 2 The BMJ, London, UK; 3 Is-design, International Institute for Information design (IIID), Vienna, Austria; 4 Faculty of Medicine and Health, The University of Sydney, Sydney, NSW, Australia; 5 Sydney School of Public Health, Faculty of Medicine and Health, The University of Sydney, Sydney, NSW, Australia; 6 Ingham Institute for Applied Medical Research, South Western Sydney Clinical School, University of New South Wales, Sydney, NSW, Australia; 7 Department of Physical Therapy, The University of British Columbia, Vancouver, British Columbia, Canada; 8 Institute for Evidence-Based Healthcare, Faculty of Health Sciences and Medicine, Bond University, Robina, Queensland, Australia

**Keywords:** evidence-based practice

## Abstract

People often use infographics (also called visual or graphical abstracts) as a substitute for reading the full text of an article. This is a concern because most infographics do not present sufficient information to interpret the research appropriately and guide wise health decisions. The Reporting Infographics and Visual Abstracts of Comparative studies (RIVA-C) checklist and guide aims to improve the completeness with which research findings of comparative studies are communicated and avoid research findings being misinterpreted if readers do not refer to the full text. The primary audience for the RIVA-C checklist and guide is developers of infographics that summarise comparative studies of health and medical interventions. The need for the RIVA-C checklist and guide was identified by a survey of how people use infographics. Possible checklist items were informed by a systematic review of how infographics report research. We then conducted a two-round, modified Delphi survey of 92 infographic developers/designers, researchers, health professionals and other key stakeholders. The final checklist includes 10 items. Accompanying explanation and both text and graphical examples linked to the items were developed and pilot tested over a 6-month period. The RIVA-C checklist and guide was designed to facilitate the creation of clear, transparent and sufficiently detailed infographics which summarise comparative studies of health and medical interventions. Accurate infographics can ensure research findings are communicated appropriately and not misinterpreted. By capturing the perspectives of a wide range of end users (eg, authors, informatics editors, journal editors, consumers), we are hopeful of rapid endorsement and implementation of RIVA-C.

**WHAT IS ALREADY KNOWN ON THIS TOPIC**

**WHAT THIS STUDY ADDS**

- We developed the 10-item Reporting Infographics and Visual Abstracts of Comparative studies (RIVA-C) checklist and guide, using a two-stage modified Delphi process, to facilitate the creation of clear and sufficiently detailed infographics summarising comparative studies of health and medical interventions.

**HOW THIS STUDY MIGHT AFFECT RESEARCH, PRACTICE OR POLICY**

- RIVA-C could improve the completeness with which research findings are communicated and avoid research findings being misinterpreted if readers (eg, health professionals and researchers) do not refer to the full-text article.

## Introduction

 Infographics (or ‘information graphics’) generally present information visually using a combination of text, images and data visualisations.^1^ They are popular tools to summarise health and medical research and increase the attention research receives.^1^,^5^ Many health and medical journals now publish infographics (a term synonymous with visual abstracts and graphical abstracts) alongside their articles and share them on social media.^1^ However, there are some issues with how infographics are used that highlight the need for increased attention to how infographics present research findings.

Many health professionals, researchers and patients use infographics as a substitute for reading full-text articles, believe infographics should be detailed enough so they do not have to read the full text, and view infographics as tools to help them save time by not having to read the full text.^6^ This may explain why some infographics decrease full-text views.^2 5 7^ People using infographics as a substitute for reading the full-text article is a concern as many infographics do not present enough of the information that is needed to interpret research appropriately and guide wise healthcare decisions. For example, a content analysis of 129 infographics from 69 health and medical journals found most infographics do not report sufficient detail for readers to understand basic study characteristics (eg, population, intervention, comparator and outcomes), potential harms of an intervention, effect size, uncertainty of effect estimates, study limitations and risk of bias.^1^ Improved detail of infographics may avoid these limitations and increase their usefulness.

There is limited rigorously developed guidance on how to appropriately report research and its findings using infographics. Some guidelines only provide recommendations for design and formatting^8^ and others were not developed using a rigorous process.^9 10^

## Aim and scope

The aim of the Reporting Infographics and Visual Abstracts of Comparative studies (RIVA-C) checklist and guide is to facilitate the creation of clear and sufficiently detailed infographics which accurately summarise comparative studies of health and medical interventions. RIVA-C could improve the completeness with which research findings of comparative studies are communicated and avoid research findings being misinterpreted if readers (eg, health professionals and researchers) do not refer to the full-text article. This article describes the development of the 10-item RIVA-C checklist and guide. It reports the methods used to reach consensus on the checklist and outlines each checklist item, including an elaboration and examples of appropriate reporting.

The primary audience for the RIVA-C checklist and guide is developers of infographics which summarise comparative studies of health and medical interventions. This includes but is not limited to infographic designers, journal editors, researchers, health professionals and members of the public. RIVA-C is not a guide for creating graphics within a research article. To best understand how to implement the checklist recommendations, we encourage people to read the explanation and examples document (online supplemental file 1).

## Methods

Development of the checklist and guide was led by an international Steering Group (led by JZ) consisting of information design experts (VE, WS-T and CW), individuals who produce infographics for journals (WS-T; Infographics Editor at The BMJ), individuals with experience in developing reporting guidelines (TH; led the development of the Template for Intervention Description and Replication [TIDieR] checklist^11^), experts in clinical research methodology (CM, ME, IH, GF, JZ and MO), editors of journals who publish infographics (ME and CA), authors who have published or developed infographics (JZ, GF, ME and IH), experts in health communication/health literacy (KM) and health professionals (AG and IH). The checklist and guide project was prospectively registered on the Enhancing the QUAlity and Transparency Of health Research (EQUATOR) Network website^12^ and developed according to the Guidance for Developers of Health Research Reporting Guidelines.^13^

The need for a checklist and guide was identified by our review of 129 infographics that summarised comparative studies of health and medical interventions, finding potential checklist items that were infrequently reported (eg, potential harms of an intervention, measures of precision, risk of bias).^1^ The steering group used these findings to develop a draft checklist (20 items that could feasibly be incorporated) for the first round of a two-round, modified Delphi survey (online supplemental file 2).

Delphi participants (n=92) had diverse and overlapping professional backgrounds: health professionals (70%), researchers (61%), methodologists (11%), journal editors (9%), infographic designers (8%), statisticians (7%), patients or members of the public (3%) and policy-makers (1%) (see online supplemental file 3 for participant characteristics). Participants rated each proposed item with the following response options: omit, possibly include, desirable and essential. For an item to reach consensus, >66% of participants needed to rate it as either of the upper two response options (desirable or essential). This threshold was based on previous studies that developed guidelines.^14 15^ Participants also provided comments on each item which were used to refine the wording of items reaching or almost reaching consensus, develop new items and refine the scope of the checklist for the second round. Item ratings from the round 1 survey and the steering group’s decision on each item can be found in online supplemental file 3.

The steering group added an ‘explanation and examples’ section to each potential checklist item for the round 2 survey (online supplemental file 4), which was completed by 68 participants (74% of round 1 respondents). Reworded items of those that almost reached consensus in round 1 were included in the draft checklist if the upper two response options (desirable or essential) were rated by more than 66% of participants.^14 15^ Reworded items of those which had a clear consensus to include in round 1 were accepted if >50% of participants were satisfied with the revision. Items with clear consensus to exclude in the round 1 survey were reincluded if >50% wanted them to be reincluded. Item ratings from the round 2 survey and the steering group’s decision on each item can be found in online supplemental file 3.

An online consensus meeting was held with members of the steering group in February 2023 to discuss the findings from the round 2 survey and refine the RIVA-C checklist and guide). The RIVA-C checklist and guide was then piloted and further refined by infographics editors or authors of infographics at *The BMJ*, Physiotherapy Evidence Database (PEDro—a research database of over 60 000 trials, systematic reviews and guidelines relevant to physiotherapy)^16^ and the *Journal of Physiotherapy* (#1 ranked journal in Rehabilitation and Orthopaedics) over a 6-month period. A more complete description of the methods and findings of the Delphi process can be found in online supplemental file 5.

### Patient and public involvement

The steering group included members of the public (VE and CAW), defined as those outside of academia, and health professionals who are regular consumers of infographics (ARG). We also had nine consumers with an interest in research infographics provide feedback on the checklist and guide. Feedback led to including the following statement in the guiding principles section: ‘Information requested from a checklist item should be presented in a way that the intended audience would understand’, adding lay language examples for the item about reporting treatment effects, and several minor wording changes.

## Results

The final 10-item RIVA-C checklist is sub-divided into 3 categories: (1) study characteristics; (2) results and (3) conclusion/take away message (online supplemental file 6). A more detailed explanation and examples document can be found in online supplemental file 1. This includes text and graphical examples for each item, with examples from a randomised controlled trial, systematic review and cohort study to showcase how to adhere to each item when summarising different types of studies. We have also included exemplar infographics from the *Journal of Physiotherapy* and PEDro. Neither of these entities have a dedicated infographics developer so the example infographics were produced with substantially less resources than the BMJ examples.

## Discussion

The RIVA-C checklist and guide will facilitate clear and sufficiently detailed infographics summarising comparative studies of health and medical interventions. This will increase the likelihood of research findings being communicated transparently and minimise the potential for misinterpretation of study findings. Ensuring research findings are communicated appropriately is particularly important for high-quality randomised controlled trials and systematic reviews as they can have important clinical implications. RIVA-C is extremely timely given recent evidence highlighting many people use infographics as a substitute for reading full-text articles^6^ and many infographics are missing key elements such as basic study characteristics, potential harms of an intervention, effect sizes and measures of precision,^1^ and contain evidence of spin (eg, selective reporting of positive outcomes).^17^

The RIVA-C checklist and guide should be used by anyone creating an infographic that summarises the findings of a comparative study of a health and medical intervention. This includes, but is not limited to, journal infographic designers, journal editors, researchers, health professionals, patients and policy-makers. Infographics will likely be clearer and more complete if journals, and their editors, endorse the checklist to their in-house infographic developers and recommend authors adhere to it when creating their own infographics. We recommend journals endorse RIVA-C similar to other checklists listed on the EQUATOR Network (eg, Consolidated Standards of Reporting Trials [CONSORT], Preferred Reporting Items for Systematic Reviews and Meta-Analyses [PRISMA], TIDieR). One way they can do this is by including a link to the checklist on the ‘instruction for authors’ page and including some information about the importance of appropriate reporting (or harm of inappropriate reporting) in infographics. Another option is for journals to email the RIVA-C checklist and guide to authors at the time their article is accepted for publication.

Some people may argue a checklist for reporting research findings in infographics is inappropriate because infographics are designed to draw in readers using visuals and encourage them to read the full text. In reality, the existence of an infographic often decreases full-text views,^2 5 7^ possibly because readers are using them as a substitute for reading the full text.^6^ Our extensive pilot testing of RIVA-C suggests it does not compromise visual appeal (as demonstrated by our example infographics) and is highly acceptable to infographic designers as it offers design flexibility.

Our development approach likely has some limitations. A Delphi panel with a higher proportion of infographics designers and/or journal editors may have yielded a different final set of items. Only 74% of respondents to the round 1 survey responded to the round 2 survey. Our cut-offs for including or removing a checklist item were based on commonly used, arbitrary cut-offs. We only pilot tested the checklist and did not perform a more formal evaluation of it. However, this is similar to the original publication of many other well-recognised checklists (eg, TIDieR,^11^ PRISMA,^18^ CONSORT).^19^

We believe it is crucial to evaluate the implementation of the RIVA-C checklist and guide and have planned a series of evaluation studies. This includes a quantitative study investigating whether infographics developed according to RIVA-C improve the transparent interpretation of research findings, knowledge retention and intention to change practice, and a qualitative study exploring whether infographics developed according to RIVA-C are more useful to readers than infographics developed without this guidance. Findings from this evaluation will inform the need to update or modify the checklist to increase impact. The steering group also hopes this checklist will encourage the development of checklists to improve the reporting of infographics summarising other types of research (eg, prognostic studies and diagnostic studies).

## supplementary material

10.1136/bmjebm-2023-112784 online supplemental file 1

## Data Availability

Data are available on reasonable request.
