# Supplementary material for: Development of the Reporting Infographics and Visual Abstracts of Comparative studies (RIVA-C) checklist and guide
Source: BMJ Evid Based Med. 2024 Jan 19;29(5):e112784. doi: 10.1136/bmjebm-2023-112784 (PMC11503056; doi:10.1136/bmjebm-2023-112784)
Supplement: online supplemental file 1 [file bmjebm-29-5-s001.pdf]

Supplementary File 1. The Reporting Infographics and Visual Abstracts of Comparative studies (RIVA-C) checklist and guide

Context

The RIVA-C checklist and guide is designed to improve the reporting of infographics summarising the findings of comparative studies of health and medical interventions, including retrospective observational studies, pre-post cohort studies, randomised controlled trials and systematic reviews.

- It does **not** apply to infographics summarising comparative studies using other designs (e.g. case studies, case series, cross-sectional observational studies).
- It does **not** apply to infographics summarising prognostic studies, diagnostic studies, and other types of research studies.

The scope of our checklist is limited to the content of an infographic. For guidance on design, consult a graphic designer or existing guidelines on this topic (e.g. THE 7 G.R.A.P.H.I.C. PRINCIPLES OF PUBLIC HEALTH INFOGRAPHIC DESIGN https://visualisinghealth.files.wordpress.com/2014/12/guidelines.pdf).

Guiding principles that apply to all checklist items

- These are guidelines and may not perfectly suit the needs of all infographics
- All infographics should include a way for readers to access the journal article (e.g. through a citation, DOI, URL, or QR code)
- Information requested from a checklist item may be presented using text and/or graphics
- Information requested from a checklist item may be presented as a footnote
- Information requested from a checklist item does not need to be duplicated in different sections of the infographic to satisfy the item (e.g. if the infographic presents the study population/participants in one section, it does not need to present the study population/participants in another)
- Each checklist item is accompanied by an 'Explanation and example(s)' section to help users implement the item
- Information requested from a checklist item should be presented in a way that the intended audience would understand

On the following pages, we outline the RIVA-C checklist items with accompanying explanation and examples (both text and graphical). Exemplar infographics can be found after the checklist.

Checklist item	Explanation and examples
**STUDY CHARACTERISTICS**	
**Study design**	
**1)** Present the study design.	• The infographic should clearly present the design of the study it is summarising (e.g., randomised controlled trial, systematic review, prospective cohort study).<br>• The study design does not need to be repeated if it is mentioned in the title of the infographic or as part of the study citation in the infographic.<br><br>EXAMPLE A: “Study design: Randomised controlled trial.”<br><br>EXAMPLE B: “Study design: Systematic review and meta-analysis.”<br><br>EXAMPLE C: “Population-based cohort study.”

EXAMPLE A (BMJ 2022;379:e072623)

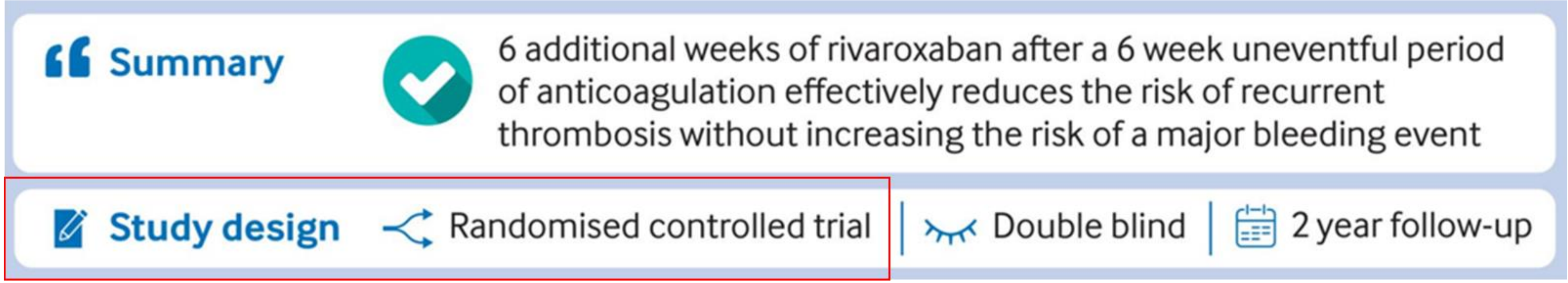

EXAMPLE B (BMJ 2021;372:m4743)

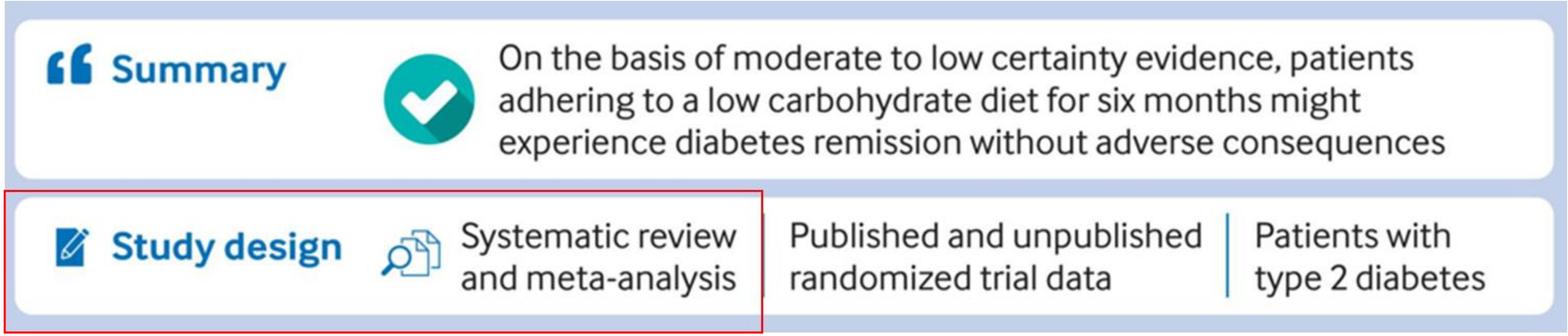

EXAMPLE C (BMJ 2022;379:e071380)

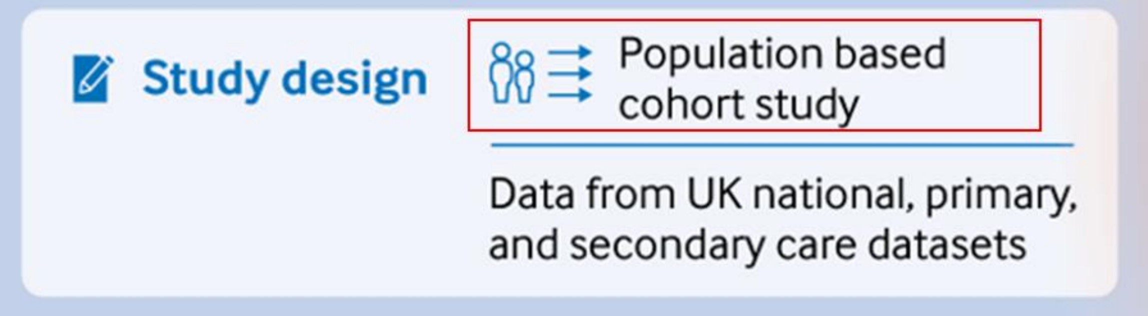

**Population**	
**2)** Present the population/participants, sample size and important characteristics describing the population/participants.	• The infographic should clearly present the population/participants and characteristics important to understanding the population/participants and interpreting the results (e.g., sample size, diagnosis, age, gender, socioeconomic status, symptom duration, study setting, country).<br>• Infographics summarising randomised controlled trials or non-randomised studies should present the number of participants randomised/enrolled (overall and for each group). Infographics summarising single-group studies should present the number of participants enrolled in the study. Infographics summarising systematic reviews should present the number of studies included and number of participants from these studies who were randomised/enrolled (overall and for each group, if feasible).<br><br>EXAMPLE A: “448 people with symptomatic isolated distal deep vein thrombosis.”<br><br>EXAMPLE B: “1357 participants with type 2 diabetes, primarily overweight and obese. Age range was 47 to 67 years.”<br><br>EXAMPLE C: “Cohort 1: 1,252 patients starting GLP-1 receptor agonists and 14,259 starting sulfonylureas. Cohort 2: 8,731 patients starting DPP-4 inhibitors and 18,204 starting sulfonylureas. Cohort 3: 2,956 patients starting SGLT-2 inhibitors and 10,841 starting sulfonylureas. Mean age ranged from 66-69 years old.”

EXAMPLE A

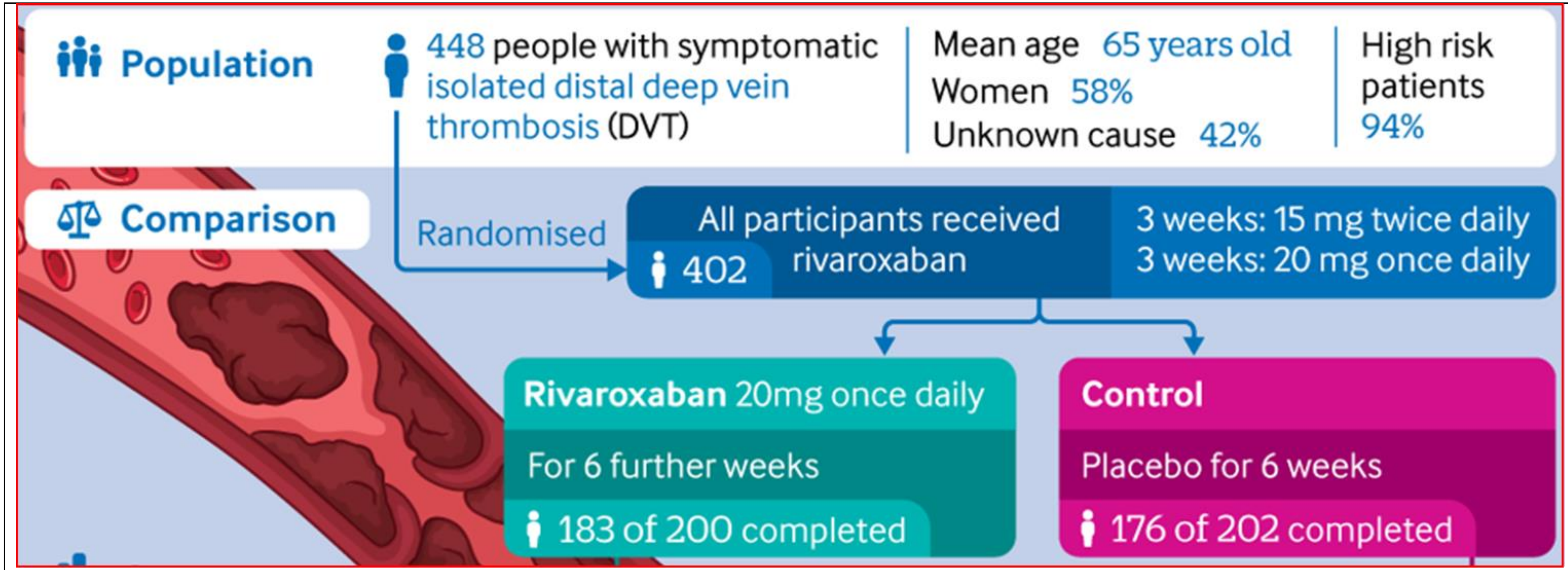
Population
448 people with symptomatic isolated distal deep vein thrombosis (DVT)
Mean age 65 years old
Women 58%
Unknown cause 42%
High risk patients 94%
Comparison
Randomised
402
All participants received rivaroxaban
3 weeks: 15 mg twice daily
3 weeks: 20 mg once daily
Rivaroxaban 20mg once daily
For 6 further weeks
183 of 200 completed
Control
Placebo for 6 weeks
176 of 202 completed

EXAMPLE B

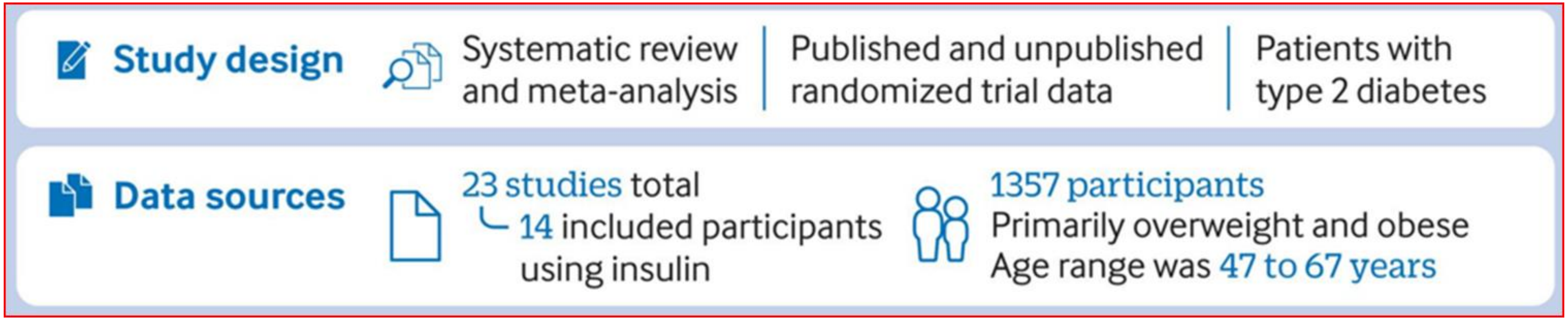

EXAMPLE C

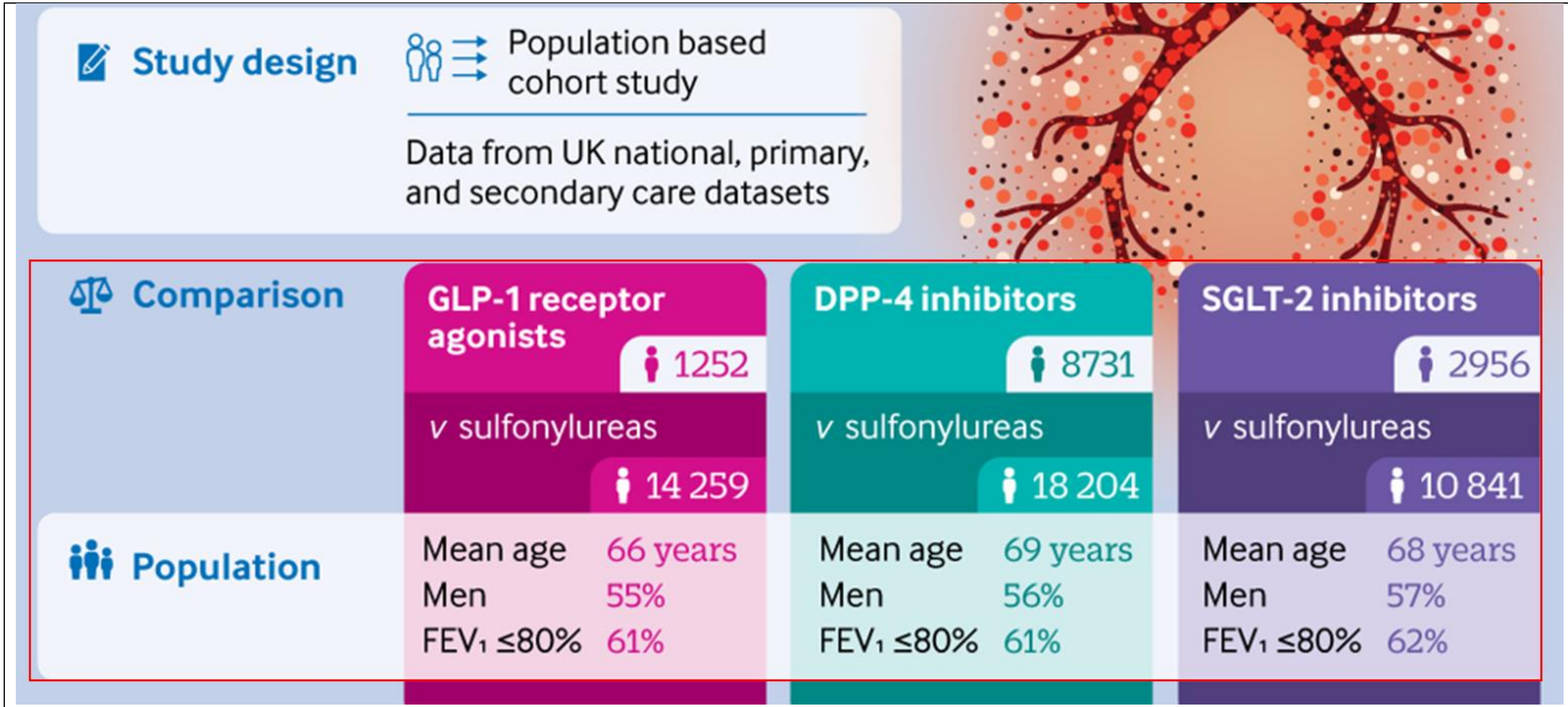

**Intervention and comparator**	
**3)** Present the intervention(s) and comparator(s) and important characteristics describing them.	• The infographic should clearly present the intervention(s) and comparator(s) (e.g., placebo, no treatment, other treatments). It should also present characteristics important to understanding the intervention(s) and

	comparator(s) and interpreting the results (e.g., drug type and dose, intervention duration, who delivered the intervention).<br>• Some studies will not have a comparator and only need to present the above information for the intervention.<br><br>EXAMPLE A: "Rivaroxaban, 20mg once daily for 6 weeks vs. Placebo for 6 weeks."<br><br>EXAMPLE B: "Low and very low carbohydrate diets vs. control (mostly low-fat diets)."<br><br>EXAMPLE C: "New user cohorts of patients starting the study drugs (GLP-1 receptor agonists, DPP-4 inhibitors, or SGLT-2 inhibitors) vs. sulfonylureas (comparison)."

EXAMPLE A

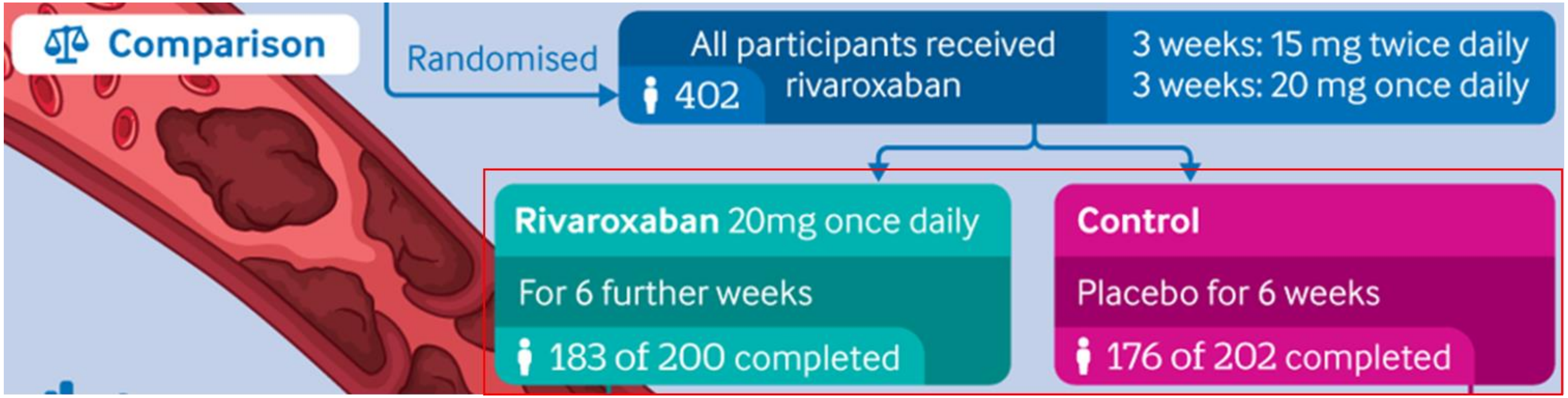

EXAMPLE B

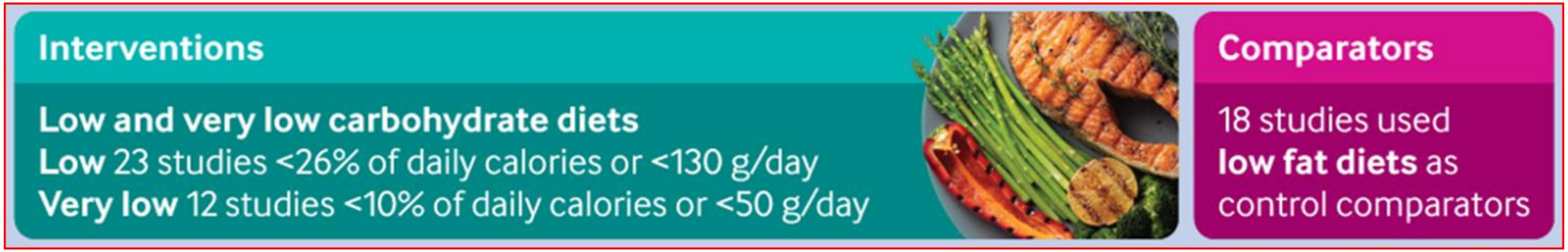

EXAMPLE C

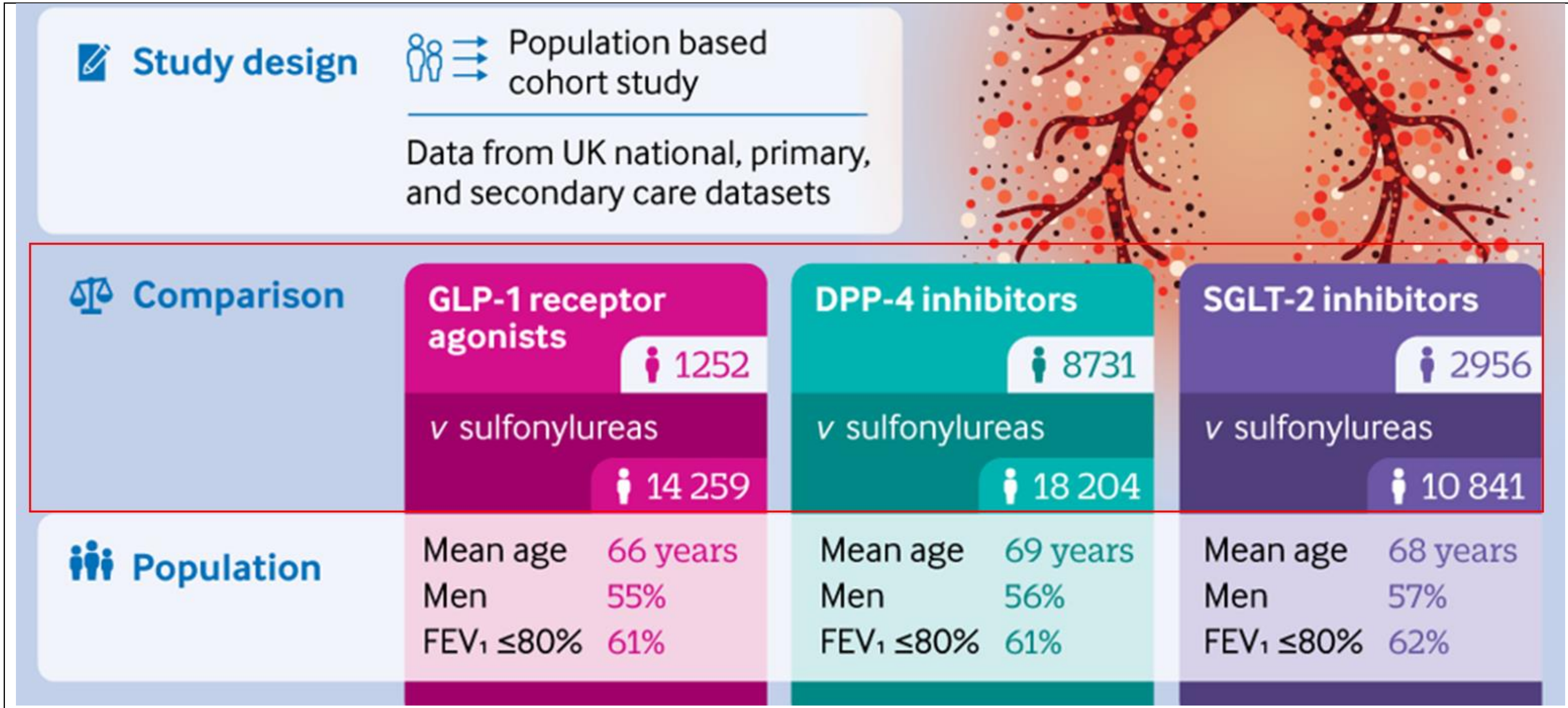

**Outcomes**	
**4)** Present and clearly label the primary outcome(s), including the scale, units and time point(s).	• The infographic should clearly present the primary outcome(s) (e.g., mortality, pain), including the scale (e.g., 0 worst – 100 best), units (e.g., mmHg), and time point(s) of assessment, if relevant.<br>• Presenting secondary outcomes is optional.<br>• If presenting primary and secondary outcomes, clearly label which outcomes are primary to reduce the risk of selective reporting.<br>• If the study did not nominate a primary outcome, make this clear in the infographic (e.g., as a footnote).

	EXAMPLE A: "The primary outcome was a composite of the presence of isolated distal DVT, proximal DVT and pulmonary embolism"<br><br>EXAMPLE B: "Primary outcomes included remission, not using diabetes medication, adverse events, HbA_{1c} (%), and weight change (kg)."<br><br>EXAMPLE C: "Severe exacerbation of chronic obstructive pulmonary disease was the primary outcome."

EXAMPLE A

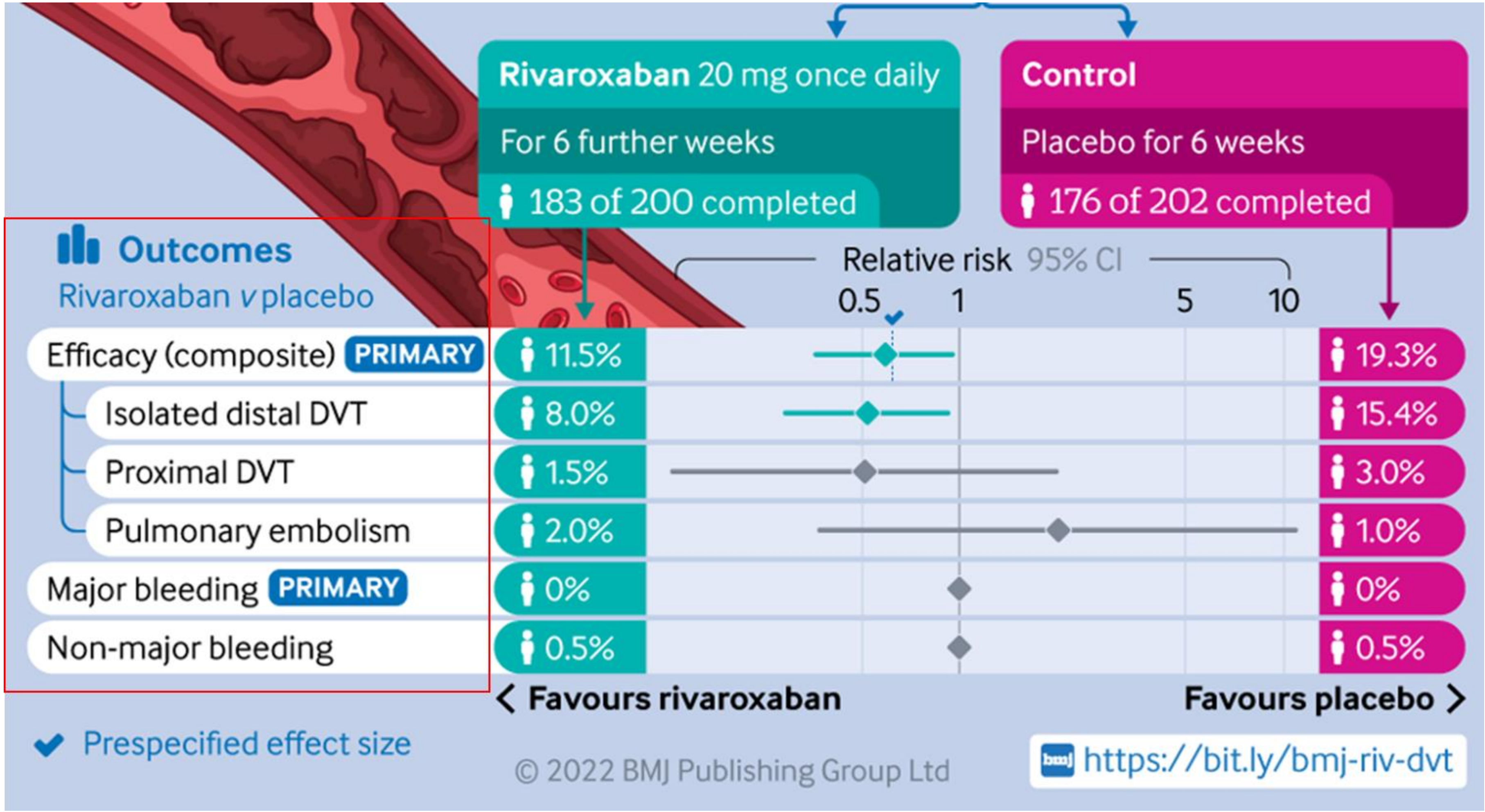

EXAMPLE B

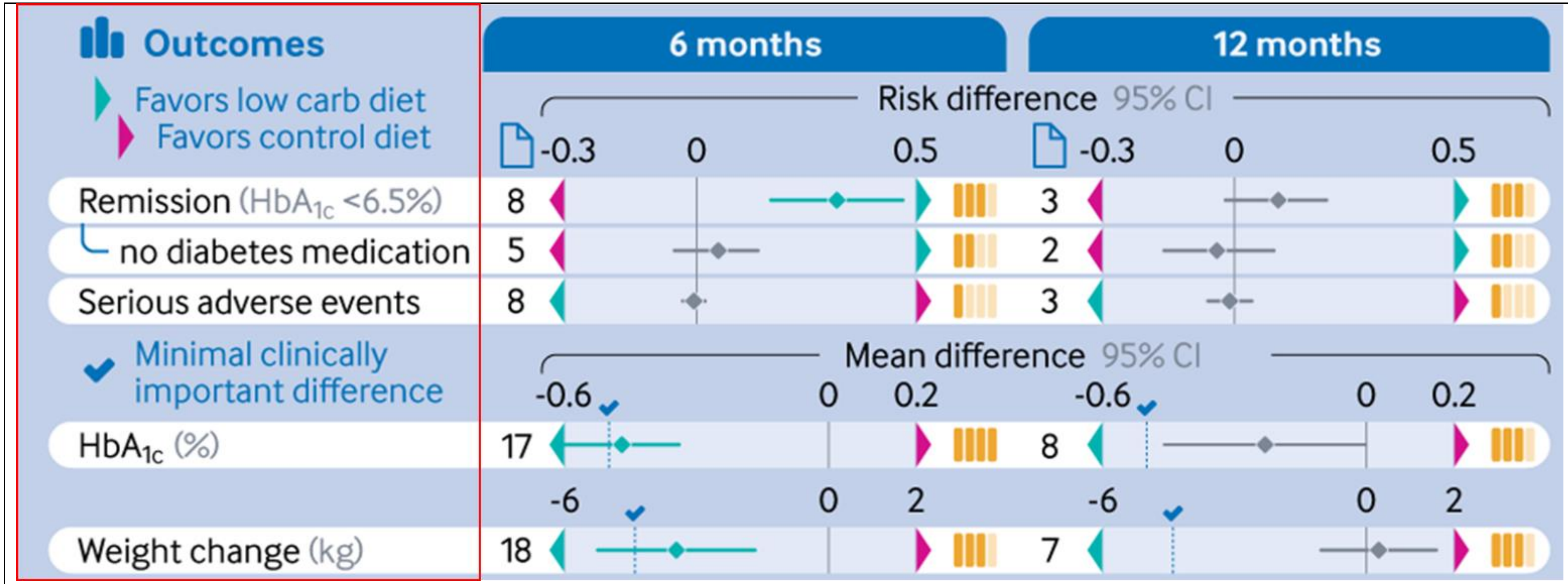
Outcomes
Favors low carb diet
Favors control diet
6 months
12 months
Risk difference 95% CI
-0.3
0
0.5
Remission (HbA1c <6.5%)
no diabetes medication
Serious adverse events
Minimal clinically important difference
Mean difference 95% CI
-0.6
0
0.2
HbA1c (%)
-6
0
2
Weight change (kg)
8
5
8
17
18
3
2
3
8
7

EXAMPLE C

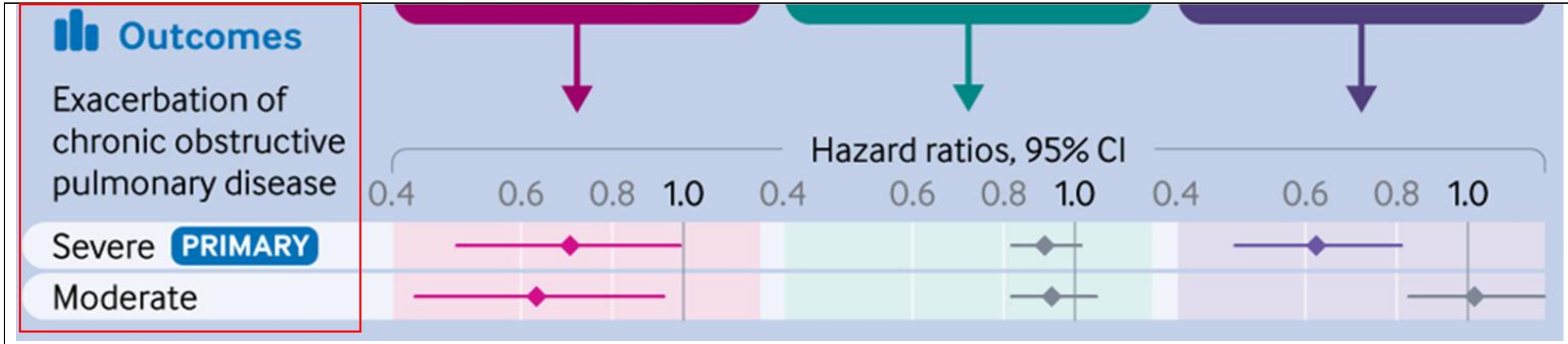	
**RESULTS**	
**How much it helps and how certain we are**	
**5)** Present between-group effects with measures of precision (e.g., mean difference and 95% CI), using absolute effects where possible, to demonstrate the effect (or lack thereof) of the intervention on the primary outcome(s) and the certainty of the effect.	• The infographic should clearly present the size (and certainty) of the effect on the primary outcome(s) using point estimates and measures of precision for between-group differences (e.g., Risk Difference or Mean Difference with 95% Confidence Intervals). Between-group differences are differences in outcomes between the intervention and control group(s) and are preferred to within-group changes (e.g., change from baseline to post-intervention). Within-group changes produce a biased effect of the intervention for several reasons (e.g., doesn't control for natural history of a disease, regression to the mean, etc.).<br>• When there isn't a comparator, the infographic should clearly present the size (and certainty) of the effect on the primary outcome using point estimates and measures of precision for within-group changes (e.g., Risk Difference or Mean Difference with 95% CI).<br>• The infographic should include the outcome values in each group (e.g., Mean of intervention vs. Mean of control) or at each time point where there isn't a comparator (e.g., Mean baseline vs. Mean post-intervention). However, we acknowledge this may not be feasible to include when multiple groups, outcomes or time points are presented.<br>• Absolute effects are preferred over relative effects (if available) as relative effects can make the magnitude of effect appear much greater than the absolute effects. For example, a decrease in risk from 1% to 0.5% equates

	to a 0.5% absolute decrease and 50% relative decrease. It is acceptable to present both absolute and relative effects.<br>• The number of participants analysed (or percentage drop out) in each group or at each time point should be presented so readers can compare it to the number of participants randomised or enrolled. This information may not be feasible to include when multiple groups, outcomes or time points are presented.<br>• Presenting point estimates and measures of precision for secondary outcomes is optional.<br>• Point estimates and measures of precision can be presented using lay language.<br><br>EXAMPLE A: "Recurrent venous thromboembolism (composite): 23 (11%) in rivaroxaban arm vs. 39 (19%) in placebo arm (relative risk 0.59, 95% CI: 0.36 to 0.95, NNT 13, 95% CI: 7-126)."<br><br>LAY LANGUAGE EXAMPLE A: "Rivaroxaban reduces risk of blood clot from 19% down to 11%. That makes a blood clot 41% less likely with rivaroxaban, with 95% CI from 5% less to 64% less."<br><br>EXAMPLE B: "Low carb diets achieved higher rates of diabetes remission (57% vs. 31%; risk difference 0.32, 95% CI: 0.17 to 0.47; 8 studies, n=264, I^2=58%)."<br><br>LAY LANGUAGE EXAMPLE B: "A low carb diet increases the likelihood of diabetes remission from 31% up to 57%. That makes experiencing reduced signs and symptoms of diabetes 68% more likely with a low carb diet, with 95% CI from 53% more to 83% more."<br><br>EXAMPLE C: "Compared with sulfonylureas, GLP-1 receptor agonists (hazard ratio 0.70, 95% CI: 0.49 to 0.99), DPP-4 inhibitors (hazard ratio 0.91, 95% CI: 0.82 to 1.02) and SGLT-2 inhibitors (hazard ratio 0.62, 95% CI: 0.48 to 0.81) were associated with a decreased risk of severe exacerbation."<br><br>LAY LANGUAGE EXAMPLE C: "When compared to people taking sulfonylureas (the oldest type of oral diabetes medication), the risk of experiencing severe worsening of chronic lung disease was 30% lower in people taking GLP-1 receptor agonists (a diabetes injection), 9% lower in people taking gliptins (oral diabetes medication) and 38% lower in people taking SGLT-2 inhibitors (oral diabetes medication)."

EXAMPLE A

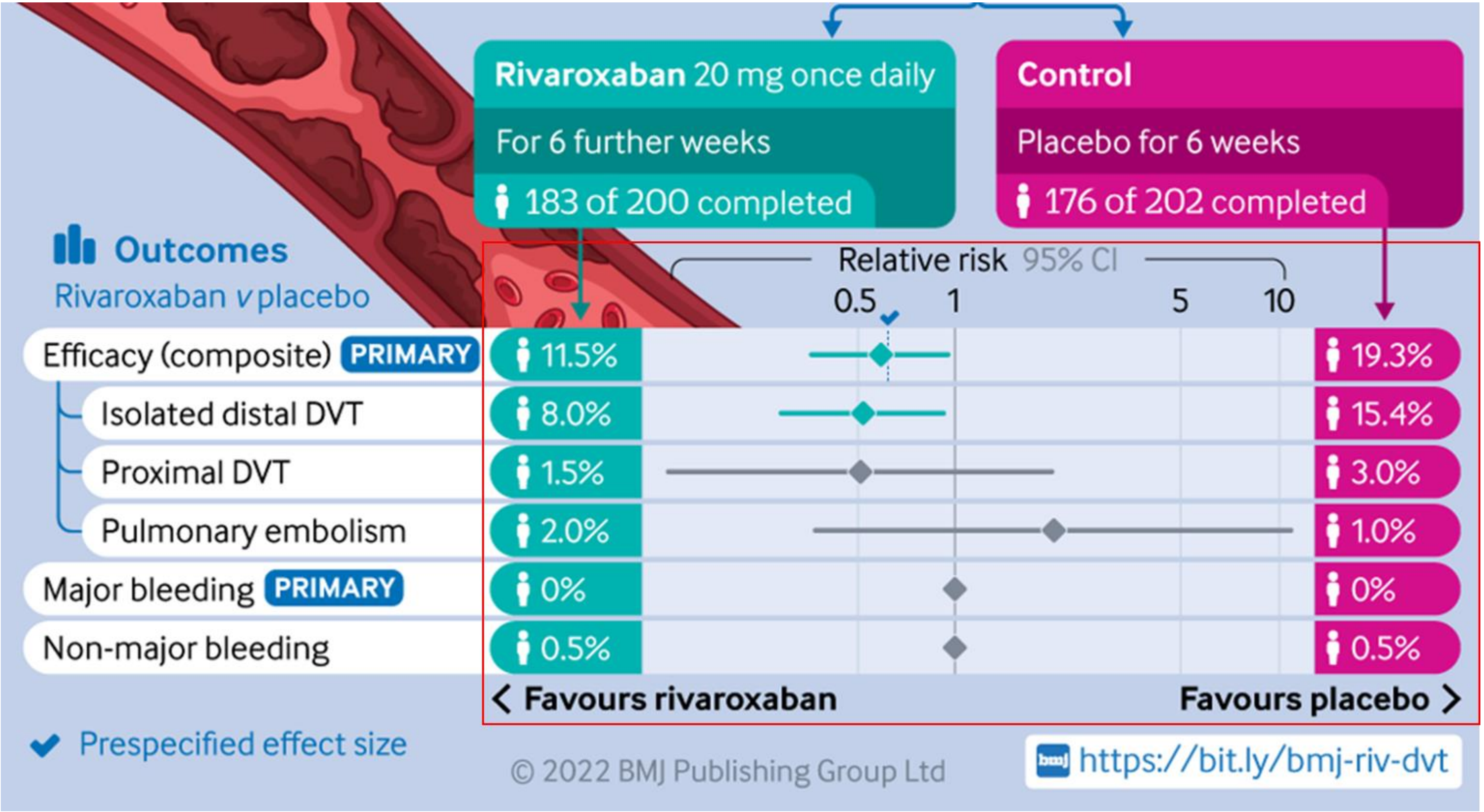

EXAMPLE B

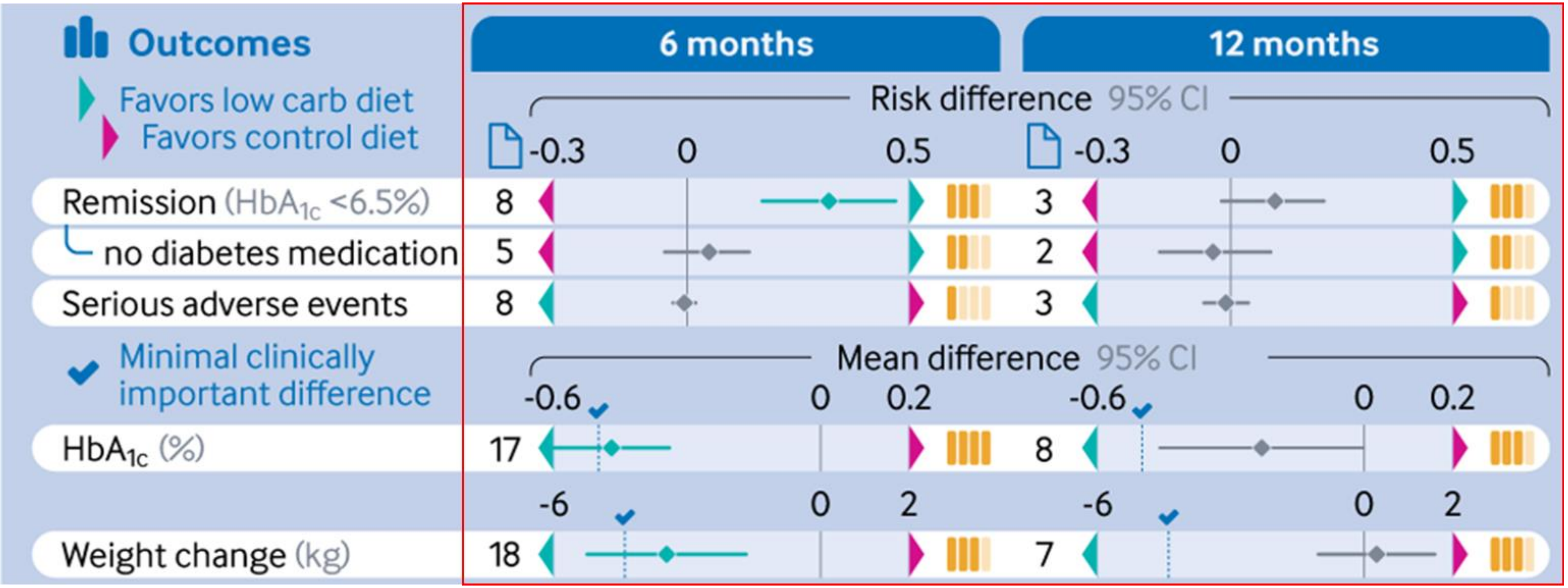
Outcomes
Favors low carb diet
Favors control diet
6 months
12 months
Risk difference 95% CI
-0.3
0
0.5
Remission (HbA1c <6.5%)
no diabetes medication
Serious adverse events
Minimal clinically important difference
Mean difference 95% CI
-0.6
0.2
HbA1c (%)
-6
2
Weight change (kg)

EXAMPLE C

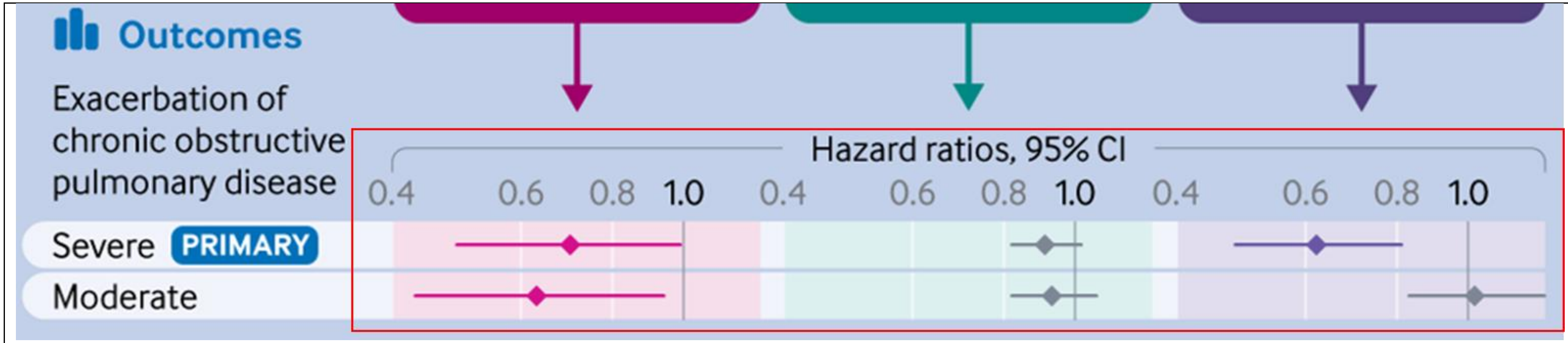

**How important are the effects**	
**6)** When possible, present the magnitude of between-group effects for the primary outcome(s) in relation to justifiable thresholds for clinical importance.	• The infographic should highlight whether the between-group effects of the intervention on the primary outcome(s) are clinically important, if justifiable thresholds exist. Justifiable thresholds are usually pre-specified by the authors (e.g. in the sample size calculation).<br>• This information can be integrated into the presentation of results (e.g. dotted line on a graph).

EXAMPLE A

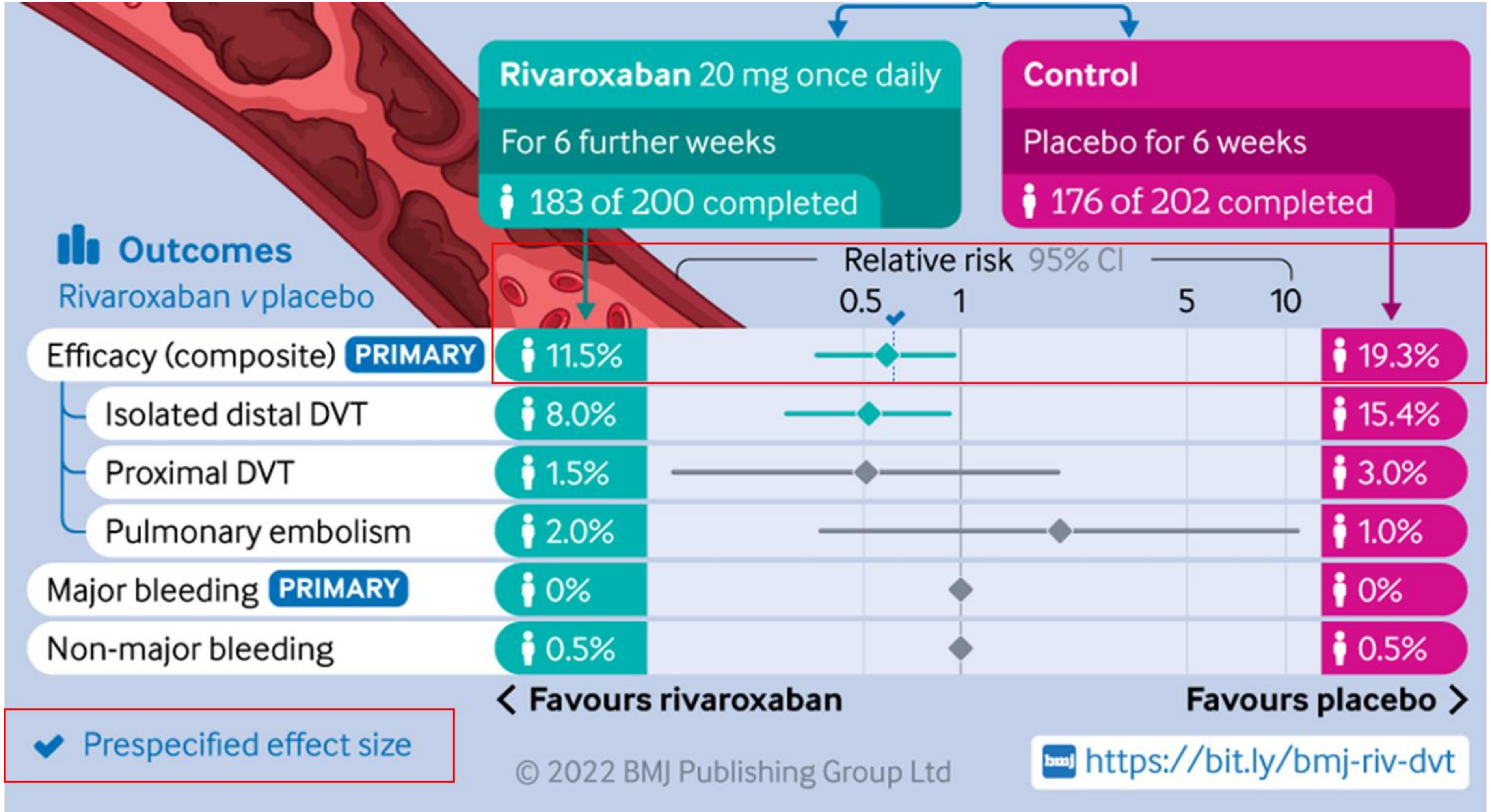

EXAMPLE B

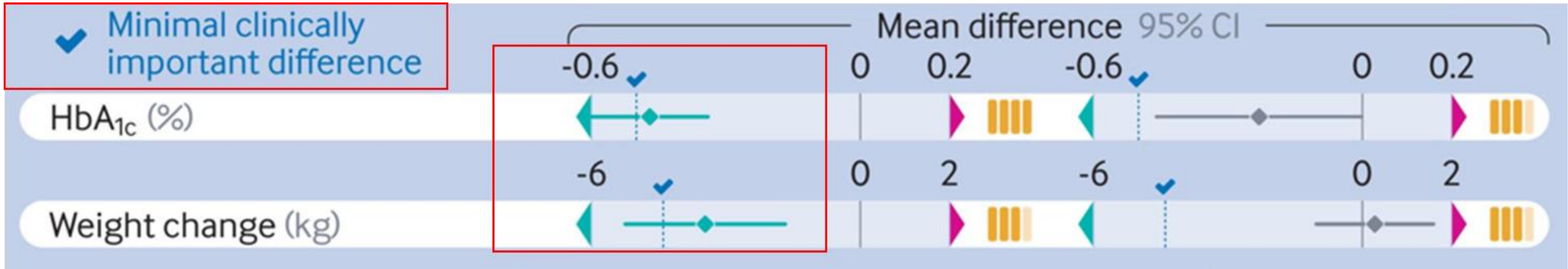

EXAMPLE C

N/A

**Whether it harms**	
**7)** Present the frequency of serious adverse events in each group and some examples of the most common serious adverse events if possible	• The infographic should clearly present the frequency of serious adverse events in each group (e.g., <u>serious adverse events</u>: control = 10% vs. intervention = 5%), and some examples of the most common serious adverse events (e.g., <u>pulmonary embolism</u>: control = 5% vs. intervention = 2%).<br>• If a study does not report the overall frequency of serious adverse events in each group, adverse events can be reported in different ways (e.g., primary safety outcome in each group, all adverse events in each group, examples of common adverse events in each group or combined).<br>• Presenting the frequency of minor adverse events in each group and some examples of the most common minor adverse events is optional, unless it is important to understanding the safety of an intervention.<br>• The infographic should highlight when a study did not report adverse events (despite measuring them), when a study did not measure them, or when no serious adverse events occurred.<br><br>EXAMPLE A: “No major bleeding events occurred.”

	EXAMPLE B: “Low carb diets did not increase total adverse events (risk difference 0.04, 95% CI: –0.01 to 0.08; 9 studies, n=423; GRADE=very low) or serious adverse events (risk difference 0.00, 95% CI: –0.03 to 0.02; 8 studies, n=448; GRADE=low).”<br><br>EXAMPLE C: “This study did not measure adverse events.”

EXAMPLE A

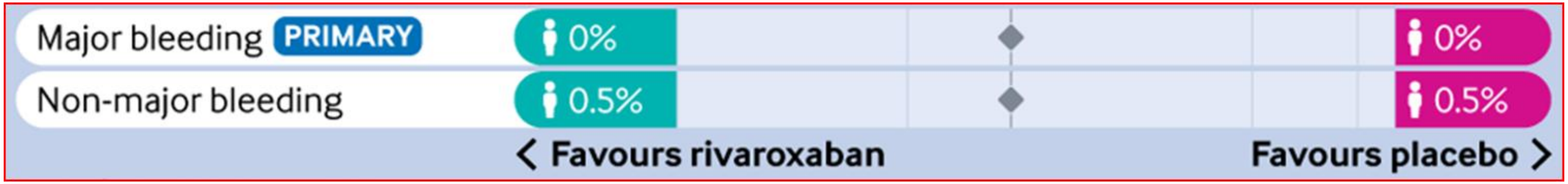

EXAMPLE B

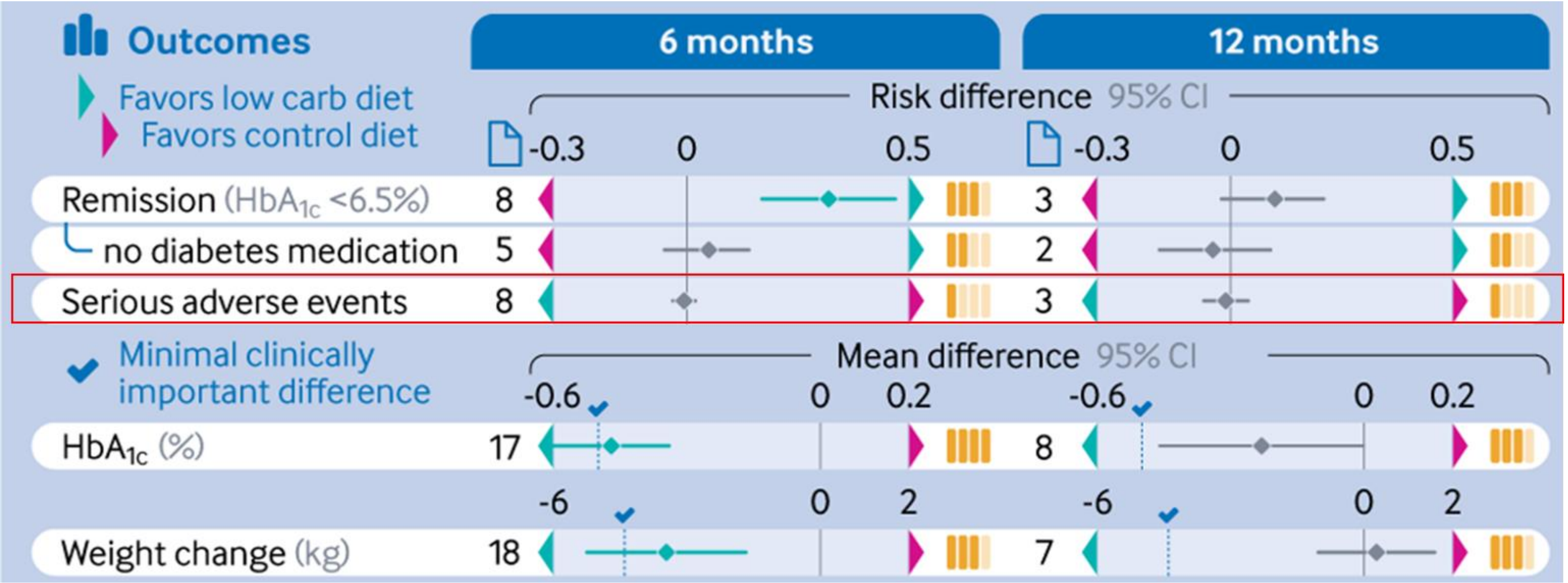

EXAMPLE C

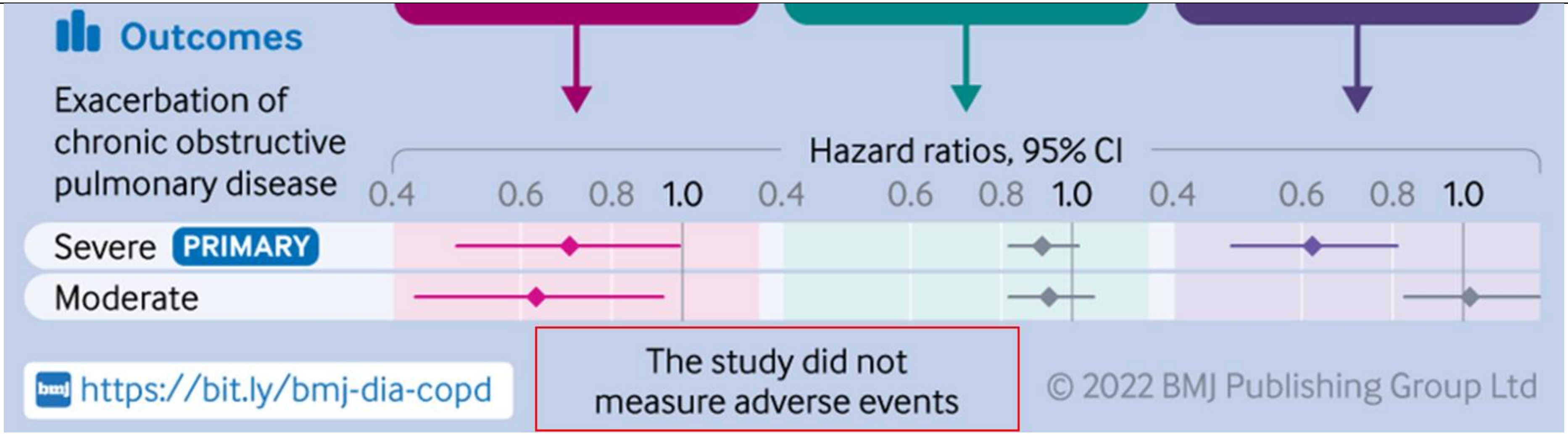

**Certainty of evidence (applicable to systematic reviews)**	
**8)** Present the certainty of evidence for all effects presented in the infographic.	• For all outcomes for which effects are reported in the infographic, the certainty of evidence should be reported also (if certainty was assessed in the original paper). If certainty of evidence was not assessed in the original paper, make this clear in the infographic (e.g., as a footnote).<br>• Presenting the certainty of evidence will allow readers to understand how certain they can be of the findings presented in the infographic or whether more research is needed.<br><br>NOTE: The Grading of Recommendations, Assessment, Development and Evaluations (GRADE, https://bestpractice.bmj.com/info/toolkit/learn-ebm/what-is-grade/) is one method for assessing certainty of evidence with certainty rated as high, moderate, low or very low.<br><br>EXAMPLE B: “No increase in total adverse events from low carb diets (risk difference 0.04, 95% CI: –0.01 to 0.08; 9 studies, n=423; GRADE=very low).”

EXAMPLE A

N/A

EXAMPLE B

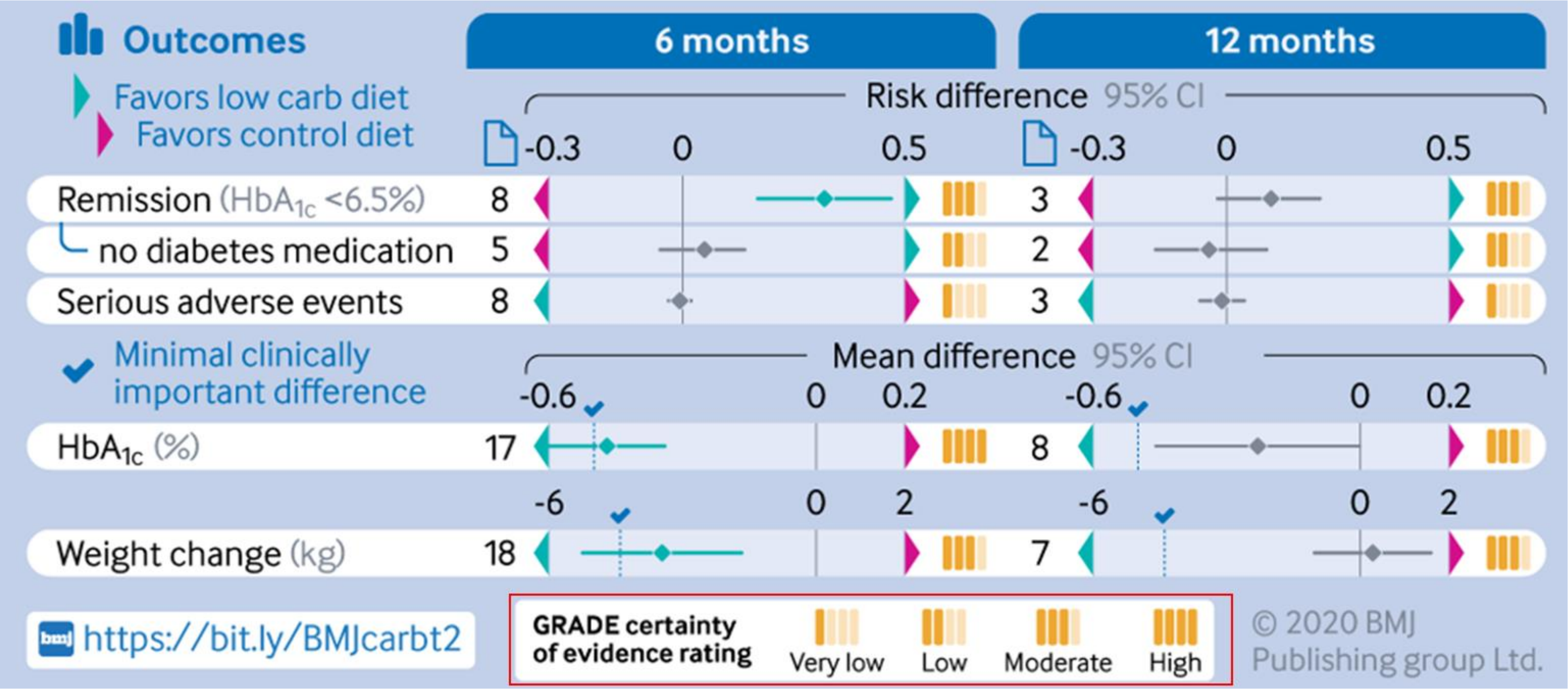

EXAMPLE C

N/A	
**CONCLUSION/TAKE AWAY MESSAGE**	
**Directness**	
**9)** When including a conclusion or take away message, ensure it is appropriate to the study population, intervention, comparator, and outcome.	• A conclusion or take away message that is appropriate to the study population, intervention, comparator, and outcomes will ensure findings are not over-generalised.<br>• A conclusion or take away message may not be necessary if other sections of the infographic present similar information.<br><br>EXAMPLE A: "6 additional weeks of rivaroxaban after a 6-week uneventful period of anticoagulation effectively reduces the risk of recurrent thrombosis without increasing the risk of a major bleeding event."<br><br>EXAMPLE B: "On the basis of moderate to low certainty evidence, patients adhering to a low carbohydrate diet for six months might experience diabetes remission without adverse consequences."<br><br>EXAMPLE C: "GLP-1 receptor agonists and SGLT-2 inhibitors, but not DPP-4 inhibitors, were associated with a lower risk of severe exacerbations compared with sulfonylureas in patients with chronic obstructive pulmonary disease and type 2 diabetes."

EXAMPLE A

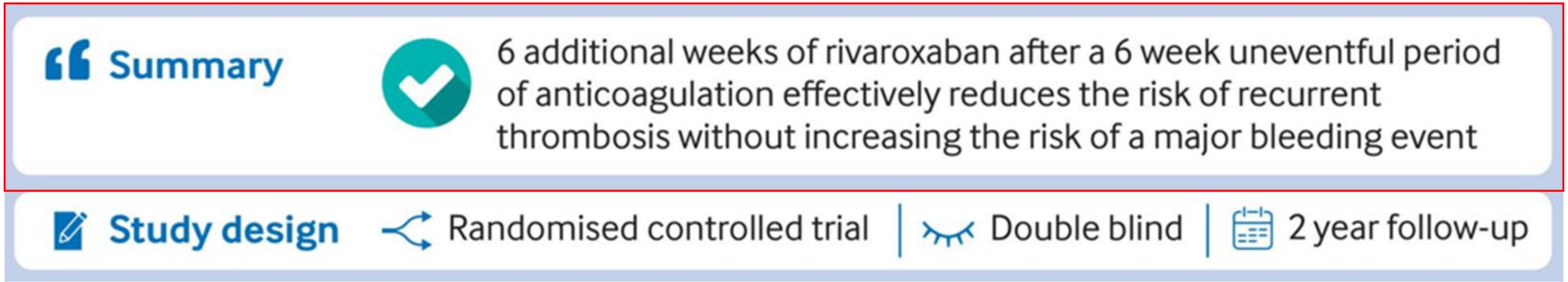

EXAMPLE B

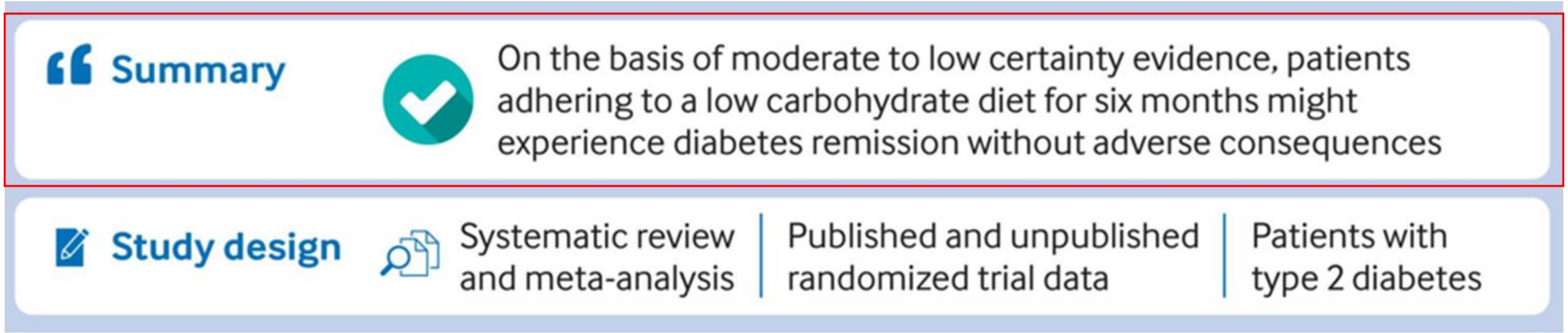

EXAMPLE C

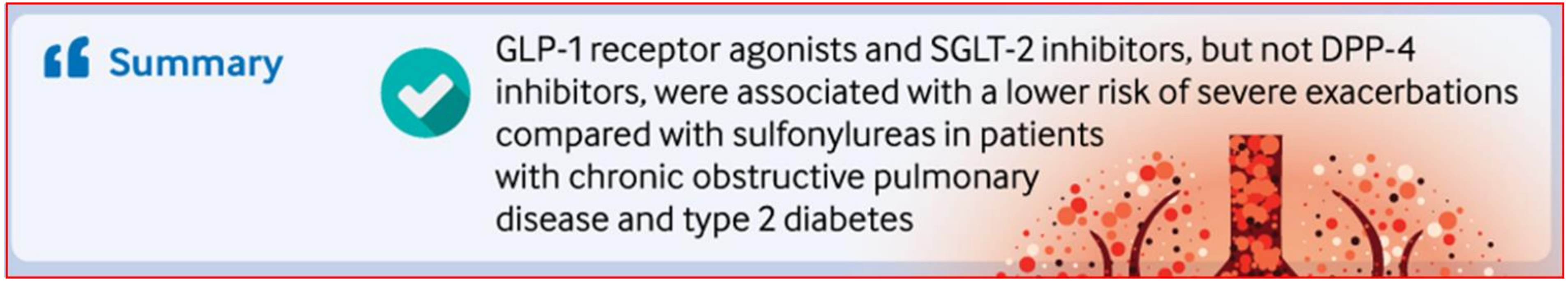

**Primary outcome**	
**10)** When including a conclusion or take away message, ensure it focuses on the primary outcome(s) and	• A conclusion or take away message that focuses on the primary outcome(s) will reduce selective reporting of statistically significant results. Acknowledging potential harms of the intervention, as compared to the comparator (if this data is available), will allow readers to weigh up efficacy and safety.<br>• Presenting findings from secondary outcomes is optional, with the exception of data on harms which is often a secondary outcome.

acknowledges potential harms of the intervention (as compared to the comparator).	• A conclusion/take away message may not be necessary if other sections of the infographic present similar information.<br><br>See EXAMPLES from checklist item #9.

Exemplar infographics

EXAMPLE A (BMJ 2022;379:e072623)

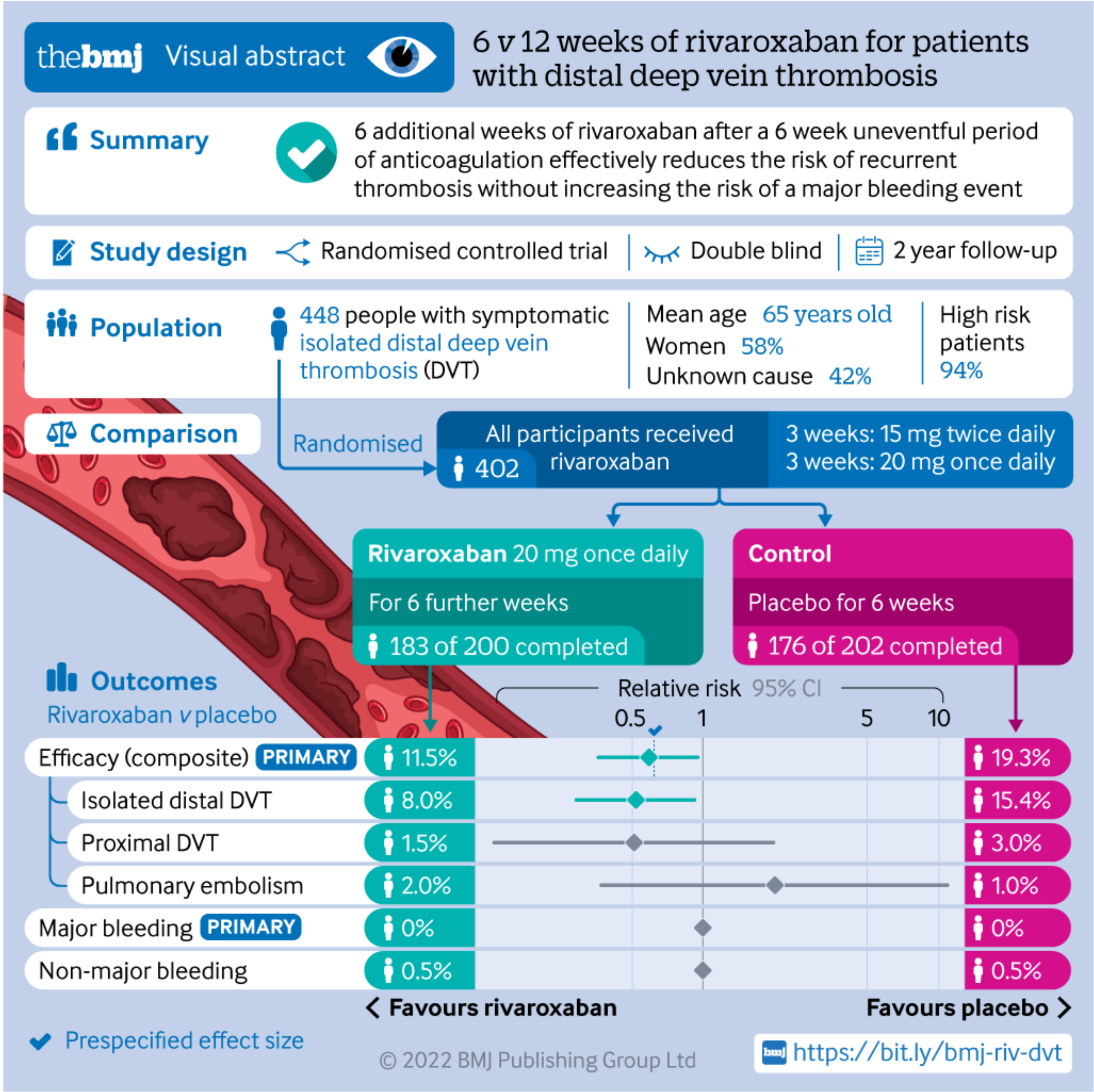

EXAMPLE B (BMJ 2021;372:m4743)

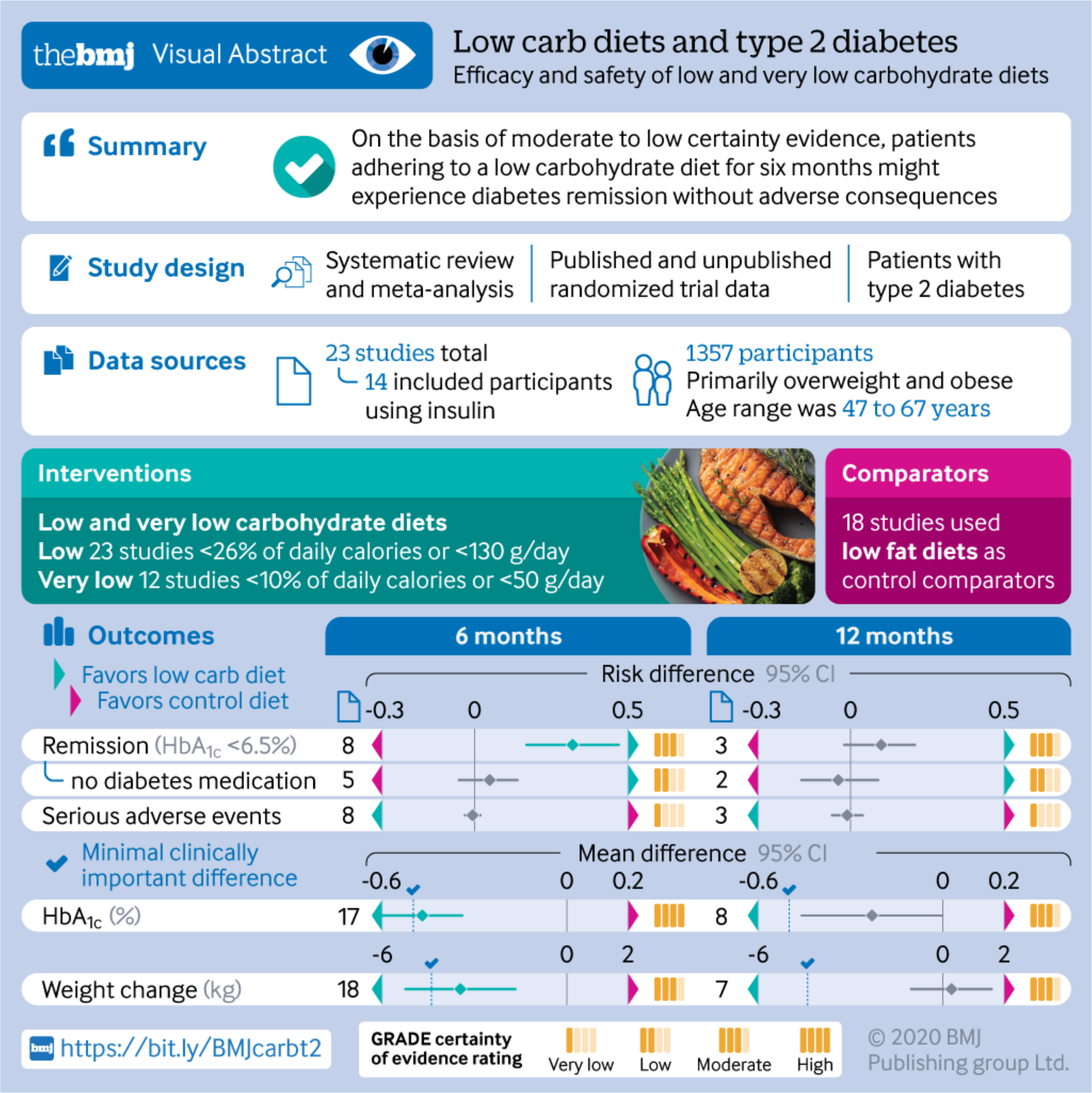

EXAMPLE C (BMJ 2022;379:e071380)

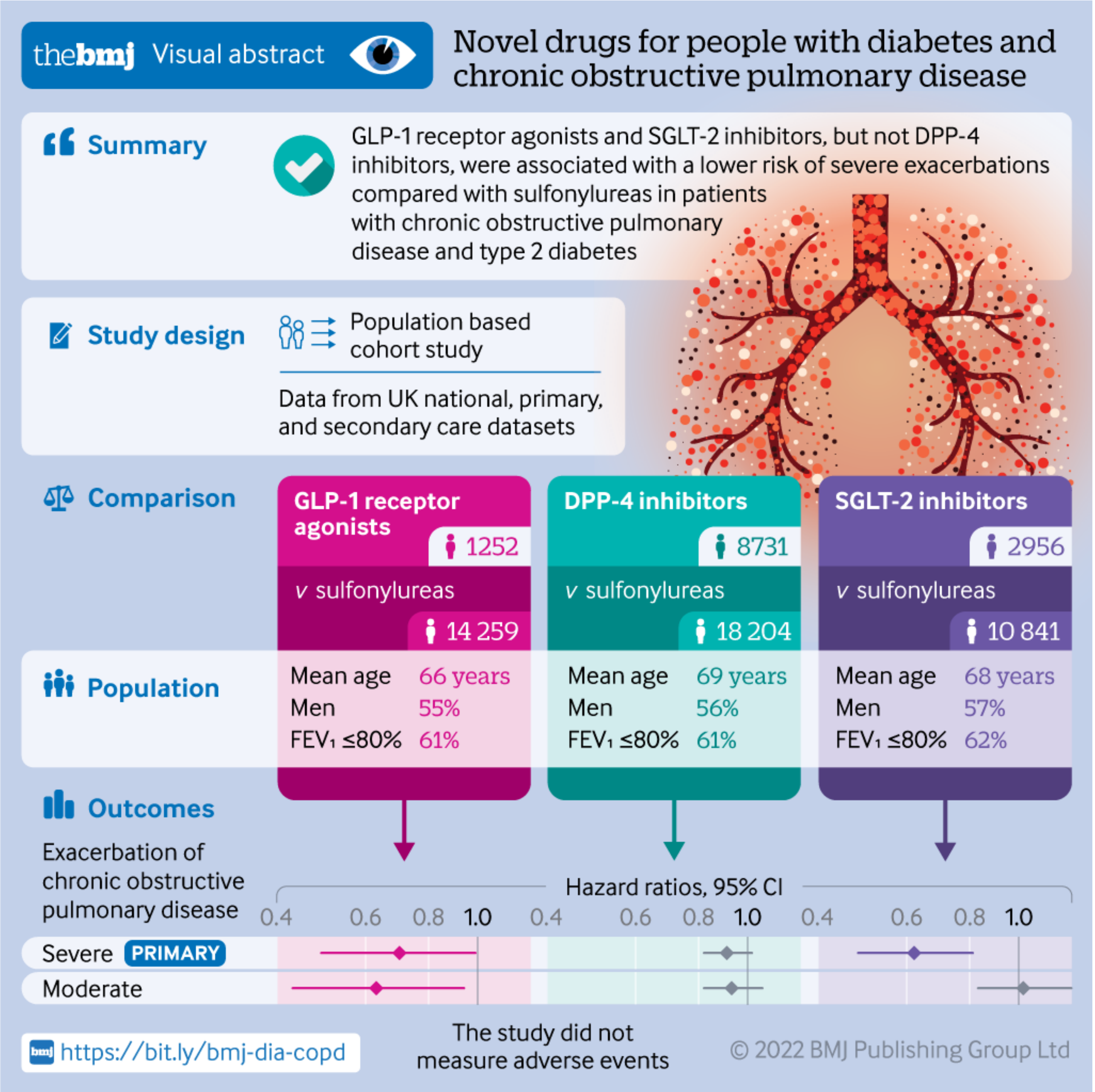

EXAMPLES FROM JOURNAL OF PHYSIOTHERAPY

Advice provides small, short-term improvements in pain and disability in non-specific spinal pain

METHODS

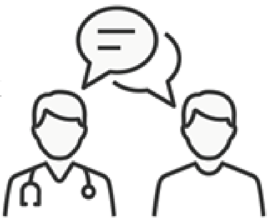

STUDY DESIGN: Systematic review of 27 randomised trials.

POPULATION: 7,006 adults with non-specific back and/or neck pain with or without radiating leg/arm pain.

INTERVENTION: Advice, defined as any advice, education or information given by a healthcare professional to improve a patient's understanding of pain or appropriate management.

COMPARATOR: No advice or placebo advice.

PRIMARY OUTCOME: Short-term (> 2 weeks but ≤ 3 months) pain and disability (0 to 100 scale).

The included trials ranged from 1 to 12 sessions, 10 to 480 minutes, and using verbal, written or mixed mode of delivery.

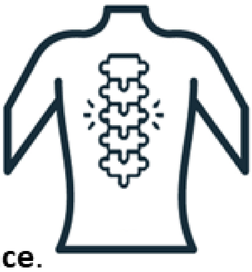

FINDINGS

PAIN
MD –8.2, 95% CI –12.5 to –3.9 (n = 2,241), **low-certainty evidence**.

DISABILITY
MD –4.5, 95% CI –7.9 to –1.0 (n = 2,579), **moderate-certainty evidence**.

ADVERSE EVENTS
Risk Diff 0.0, 95% CI -0.01 to 0.01 (n = 1,500), **moderate-certainty evidence**.

Journal of PHYSIOTHERAPY

Free full text: https://bitly.ws/VhXR
Jones CMP, et al. 2021, 67(4):263–270

Labels and advice influence perceived need for surgery in people with rotator cuff related shoulder pain, with larger effects for advice

METHODS

STUDY DESIGN: 2x2 factorial online randomised experiment.

POPULATION: 2,028 people with shoulder pain read a hypothetical scenario of a patient with rotator cuff-related shoulder pain who is given a diagnostic label and advice by a health professional.

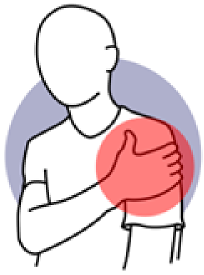

INTERVENTION: Randomised into 1 of 4 groups:

- *bursitis* label plus guideline-based advice (n = 495)
- *bursitis* label plus treatment recommendation (n = 508)
- *rotator cuff tear* label plus guideline-based advice (n = 523)
- *rotator cuff tear* label plus treatment recommendation (n = 513)

Guideline-based advice included encouragement to stay active and positive prognostic information. *Treatment recommendation* stressed that treatment is needed for recovery.

PRIMARY OUTCOME: Perceived need for surgery (0 to 10 scale), assessed immediately after reading the vignette.

FINDINGS

2,024/2,028 responses analysed (99.8%)

- Labelling as *bursitis* (versus *rotator cuff tear*) decreased perceived need for surgery (MD –0.5, 98.3% CI –0.7 to –0.2).
- *Guideline-based advice* (versus *treatment recommendation*) decreased perceived need for surgery (MD –1.0, 98.3% CI –1.3 to –0.7).

ADVERSE EVENTS: Not assessed.

NOTE: Online study; results may be different in a real-world trial.

Journal of PHYSIOTHERAPY

Free full text: https://bitly.ws/VhYc
Zadro JR, et al. 2022, 68(4):269–276

A PCP in EDs can reduce waiting and treatment times for musculoskeletal presentations, and result in more patients discharged within the 4-hour national target

METHODS

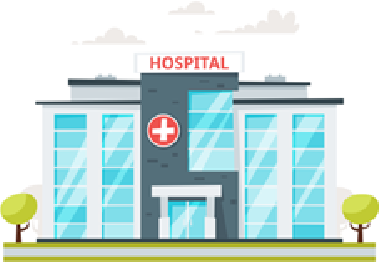

STUDY DESIGN: Prospective cohort study.

POPULATION: 13,964 patients with musculoskeletal conditions treated by 29 primary contact physiotherapists (PCP) vs. 133,668 patients matched by diagnostic codes treated by other practitioners.

Triage categories 3, 4 and 5 (less urgent) were included.

SETTING: 10 Australian emergency departments (ED).

INTERVENTION: PCPs in ED (Oct 2012 to Dec 2013).

COMPARATOR: Other practitioners in ED (Oct 2012 to Dec 2013).

OUTCOMES: Waiting time, treatment time and % discharged within 4 hours (no primary outcome specified).

FINDINGS

Being treated by PCP:

reduced wait times by 31 minutes
55 min vs. 24 min, 95% CI –32 to –30, n = 145,615

reduced treatment time by 30 minutes
148 min vs. 108 min, 95% CI –41 to –38, n = 145,613

increased % discharged within 4 hours by 18%
75% vs. 93%, 95% CI 18 to 19, n = 111,253

ADVERSE EVENTS: Not assessed.

Journal of PHYSIOTHERAPY

Free full text: https://bitly.ws/VhYj
Bird S, et al. 2016, 62(4):209-214

EFFECTS OF AEROBIC EXERCISE PERFORMED DURING PREGNANCY ON HYPERTENSION AND GESTATIONAL DIABETES

Zhang J et al. *J Sports Med Phys Fitness*. 2023;63(7):852-863.

WHAT DID THEY DO?

Study design: Systematic review of 11 randomised controlled trials.

Population: 3,165 pregnant women.

Intervention: Aerobic exercise (eg, walking on land or in water, cycling, yoga) minimum 3 days/week, 30-60 minutes, 6-40 weeks.

Comparator: Standard antenatal care and education.

Outcome: Incidence of gestational diabetes mellitus and gestational hypertension.

Most trials (8/11) had low risk of bias.

FINDINGS

Aerobic exercise vs. standard antenatal care and education led to:

- 61% less odds of gestational diabetes mellitus (95% CI 50% to 70% less likely).
- 62% less odds of gestational hypertension (95% CI 46% to 73% less likely).

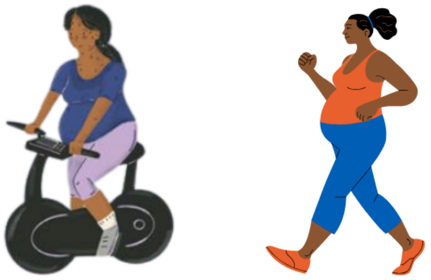

Note: Adverse events not reported. Certainty of evidence was not assessed. No primary outcome specified.

Aerobic exercise during pregnancy reduces the incidence of gestational diabetes mellitus and gestational hypertension compared to standard antenatal care.

pedro.org.au
@PEDro_database
@PEDro_database
Physiotherapy Evidence Database

PEDro
Physiotherapy Evidence Database

EXERCISE-BASED REHABILITATION PROGRAMMES FOR PULMONARY HYPERTENSION

Morris NR, et al. *Cochrane Database Syst Rev.* 2023;3(3):CD011285

WHAT DID THEY DO?

Study design: Systematic review of 14 trials (11 in the meta-analysis).

Population: 574 adults with pulmonary hypertension (462 in meta-analysis) who were medically stable.

Intervention: Supervised exercise-based rehab, run in either inpatient or outpatient settings and including both upper and lower limb exercises.

Comparator: Education or usual care with no specific exercise component.

Outcome: The primary outcome was exercise capacity, including measures such as 6MWT (distance walked in m), peak exercise capacity (peak O2 uptake mL/kg/min).

FINDINGS

Supervised exercise-based rehabilitation compared with control:

- ↑ mean six-minute walk distance by 49m, 95% CI 33 to 64; low certainty evidence.
- ↑ mean peak oxygen uptake by 2.1 mL/kg/min, 95% CI 1.6 to 2.6; low certainty evidence.
- Did not increase risk of serious adverse events (risk difference 0, 95% CI -0.03 to 0.03); moderate certainty evidence.

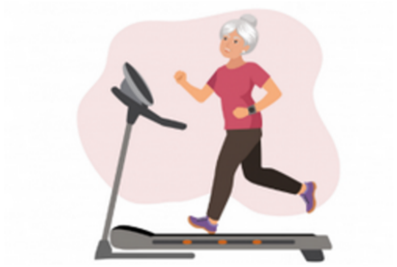

In people with pulmonary hypertension who are medically stable, supervised exercise-based rehabilitation may lead to a large increase in exercise capacity with no significant harm when compared to a non exercise-based intervention.

pedro.org.au
@PEDro_database
@PEDro_database
Physiotherapy Evidence Database

PEDro
Physiotherapy Evidence Database

EFFECT OF DIFFERENT TYPES OF EXERCISE IN ADULT SUBJECTS WITH FIBROMYALGIA

Couto N, et al. *Sci Rep*. 2022;12(1):10391

WHAT DID THEY DO?

Study design: Systematic review of 18 randomised controlled trials.

Population: 1,184 adults with fibromyalgia.

Intervention: Land based exercise (aerobic, resistance or stretching).

Comparator: Usual care.

Outcome: Pain, depression, health-related quality of life (HRQoL).

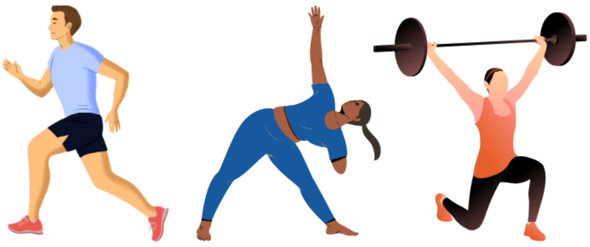

FINDINGS

Compared to usual care, exercise:

- ↓ pain (SMD=-1.3, 95% CI -1.7 to -1.0, I^2=85%).
- ↓ depression (SMD=-0.8, 95% CI -1.3 to -0.3, I^2=85%).
- ↑ HRQoL (SMD=1.0, 95% CI 1.3 to 0.6, I^2=82%).
- ↑ mental (SMD=0.5, 95% CI 0.2 to 0.8, I^2=55%) and physical (SMD=0.8, 95% CI 0.5 to 1.1, I^2=62%) components of HRQoL.

Low certainty evidence for all outcomes.

Note: Outcome scales and adverse events were not reported. No primary outcome specified.

Exercise training for people with fibromyalgia may reduce pain and depression, and improve HRQoL.

pedro.org.au
@PEDro_database
@PEDro_database
Physiotherapy Evidence Database

PEDro
Physiotherapy Evidence Database

Supplementary File 2. Round 1 survey

Welcome to the survey

Welcome to the health and medical infographics project!

Thank you for your interest.

What is the aim of this project?
Researchers at the University of Sydney are doing this project to develop a checklist of essential items to report in infographics that summarise the findings of comparative studies of health and medical interventions (e.g. randomised controlled trials, systematic reviews).

A checklist to facilitate clear, transparent, and sufficiently detailed infographics summarising comparative studies of health and medical interventions is needed to improve the accuracy with which research findings are communicated and avoid research findings being misinterpreted if consumers (e.g. health professionals, researchers) do not refer to the main paper.

To develop a checklist, we need to explore what information people consider important to include in infographics.

What does participation involve?
Participation involves completing two surveys (with the possibility of a third) between January 2022 and June 2022. The first survey is ready for you to complete. The second survey will be emailed to you at a later date.

Each survey will take approximately 15-20 minutes. Our researchers take your privacy very seriously and all responses will be anonymous. You can also exit from the survey at any time.

A Participant Information Sheet is available here. You should review and retain this information sheet before proceeding. Please read it carefully before making up your mind about taking part. If you have any questions, please get in touch with one of the research team using the phone numbers or emails listed in the information sheet.

The University of Sydney Human Research Ethics Committee has approved this study (Protocol number: 2021/723).

The next screen will ask for your consent.

Who can participate?
You must be 18 years or older and be able to read and write English to take part in this study.

We are looking for a range of people to participate including statisticians and methodologists, individuals who produce infographics for journals (e.g. Informatics Editors), policy makers, editors of journals from various fields of medicine and health, authors who have published or developed infographics, and consumers (e.g. health professionals, members of the public). Thank you for supporting this important research.

Participant Consent Form

In giving my consent I acknowledge that:

✓	I have read the Participant Information Statement and have been given the opportunity to discuss the study and my involvement in it with the researcher/s.
✓	The procedures required and time involved (including any inconvenience, risk, discomfort or side effect, and their implications) have been explained to me, and my questions about the project have been answered to my satisfaction.
✓	I understand that participation is voluntary. I am under no obligation to consent.
✓	I understand that I can withdraw from the study at any time, without providing a reason and without suffering any penalty. This will not affect my relationship with the researcher/s or university.
✓	I understand that my involvement is strictly confidential and no information about me will be used in any way that reveals my identity.
✓	I understand that data from this study may be used again for future research purposes, but that all data is strictly confidential and no information about me will be used in any way that reveals my identity.
✓	I would like the researchers to contact me to inform me about the results of the study.

- ◯ Yes, I would be happy to go on and complete the survey
- ◯ No, I would prefer not to complete the survey

I consent to the future use of any data I provide for research purposes. I understand that before the researchers can use any data I provide, they must seek additional ethics approval.

- ◯ Yes
- ◯ No

I would like the researchers to contact me to inform me about the results of the study

- ◯ Yes
- ◯ No

Demographics

Welcome to the Round 1 survey!

This survey should not take more than 15-20 minutes.

You do not have to complete the survey in one sitting. If you use the same computer or device, you can return to the survey at any time.

The survey will remain for 3 weeks.

First, some questions about you…

Please enter your email address (this is so we can contact you for the next survey and contact to you to inform you about the results of the study if you indicated you would like us to do so. Your email address will be stored separately from your responses so we cannot identify you)

Please verify your email address

Please indicate your gender

- Male
- Female
- Non-binary / third gender
- Prefer not to say

Please indicate your age

In which country were you born?

What option best describes your highest level of education?

- Primary school completed or less
- High school (not completed)
- High school (completed)
- TAFE/Trade (completed)
- University- undergraduate degree/s (completed)
- University- postgraduate degree/s e.g. Masters, PhD (completed)
- Other (please specify)

What is your employment status?

- Employed full-time
- Employed part-time
- Casual work
- Retired
- Unemployed
- Student
- Sick/disability leave
- Other (please specify)

What is your background? Please select all that apply

- ☐ Researcher (please specify the field) ______
- ☐ Statistician
- ☐ Health professional (please specify the profession) ______
- ☐ Patient or member of the public
- ☐ Methodologist
- ☐ Journal Editor (please specify the journal(s)) ______
- ☐ Policy maker
- ☐ Infographics Editor for a journal (please specify the journal(s)) ______
- ☐ Infographics designer
- ☐ Other (please specify) ______

Have you ever developed/designed (or helped develop/design) an infographic(s) summarising research (e.g. visual abstract)?

- ○ No
- ○ Yes

How many infographics have you developed/designed (or helped develop/design)?

- 1
- 2-5
- 6-20
- 21-50
- >50

How many were published (or appear) in a peer reviewed journal?

- 0
- 1
- 2-5
- 6-20
- 21-50
- >50

Have you ever developed/designed (or helped develop/design) an infographic summarising the findings of a comparative study of a health and medical intervention (e.g. randomised controlled trial, systematic review)?

- No
- Yes

How many of these infographics have you developed/designed (or helped develop/design)?

- 1
- 2-5
- 6-20
- 21-50
- >50

Delphi survey

Next, we would like you to rate and comment on a list of potential items to include in a checklist for infographics that summarise the findings of comparative studies of health and medical interventions (e.g. pre-post cohort studies, randomised controlled trials, systematic reviews).

IMPORTANT INFORMATION

We would like you to consider the following guiding principles when reviewing items for inclusion:

1. Reporting of the item should **FACILITATE** accurate interpretation of a study's findings;
2. The item is likely relevant to **ALL** infographics summarising the findings of comparative studies of health and medical interventions (e.g. pre-post cohort studies, randomised controlled trials, systematic reviews);
3. The set of items represent the **MINIMUM** that should be reported in all infographics summarising the findings of comparative studies of health and medical interventions (items are not too detailed for a 'minimum reporting guideline');
4. Adding items may **REDUCE** the clarity and visual appeal of the infographic

Please indicate whether each proposed item should be **omitted** or kept in the checklist (and whether it is considered **possible**, **desirable** or **essential**). Please provide the reason for your response in the comments section.

You will be shown 20 proposed checklist items. The final checklist may have more or less items, depending on your response.

Please rate and comment on all checklist items.

Checklist item 1

STUDY DESIGN

Include the study design (e.g. pre-post cohort study, randomised controlled trial, systematic review). Can be included in the infographic's title or study title as a citation

Item 1 of 20

- ◯ Omit
- ◯ Possibly include
- ◯ Desirable
- ◯ Essential

Please provide the reason for your above response

Checklist item 2

STUDY CHARACTERISTICS

Population

Depict the population/participants (e.g. older people with chronic low back pain) using text and/or graphics

Item 2 of 20

- ◯ Omit
- ◯ Possibly include
- ◯ Desirable
- ◯ Essential

Please provide the reason for your above response

Checklist item 3

STUDY CHARACTERISTICS

Population

Include at least one important quantitative characteristic of the population/participants (e.g. mean age, mean symptom duration)

Item 3 of 20

- ○ Omit
- ○ Possibly include
- ○ Desirable
- ○ Essential

Please provide the reason for your above response

Checklist item 4

STUDY CHARACTERISTICS

Intervention

Depict the intervention (e.g. acupuncture) using text and/or graphics

Item 4 of 20

- ◯ Omit
- ◯ Possibly include
- ◯ Desirable
- ◯ Essential

Please provide the reason for your above response

Checklist item 5

STUDY CHARACTERISTICS

Intervention

Include at least one important quantitative characteristic of the intervention (e.g. drug dose, intervention duration)

Item 5 of 20

- Omit
- Possibly include
- Desirable
- Essential

Please provide the reason for your above response

Checklist item 6

STUDY CHARACTERISTICS

Comparator

Depict the comparator (e.g. no treatment) using text and/or graphics

Item 6 of 20

- ◯ Omit
- ◯ Possibly include
- ◯ Desirable
- ◯ Essential

Please provide the reason for your above response

Checklist item 7

STUDY CHARACTERISTICS

Comparator

Include at least one important quantitative characteristic of the comparator (e.g. drug dose, intervention duration)

Item 7 of 20

- ○ Omit
- ○ Possibly include
- ○ Desirable
- ○ Essential

Please provide the reason for your above response

Checklist item 8

STUDY CHARACTERISTICS

Outcome

Depict the outcome’s construct (e.g. mortality, pain) using text and/or graphics and clearly label outcomes as primary or secondary

Item 8 of 20

- ○ Omit
- ○ Possibly include
- ○ Desirable
- ○ Essential

Please provide the reason for your above response

Checklist item 9

STUDY CHARACTERISTICS

Outcome

Describe how the primary outcome was assessed, including the scale of the assessment tool

(e.g. physical function as assessed by the SF-36, 0-100 scale)

Item 9 of 20

- ○ Omit
- ○ Possibly include
- ○ Desirable
- ○ Essential

Please provide the reason for your above response

Checklist item 10

OVERALL RESULTS OF THE STUDY

Benefits

Depict the benefits of the intervention according to the outcomes assessed (e.g. improves mortality, reduces disease reoccurrence) using text and/or graphics (i.e. do not mention benefits that were not assessed in the study)

Item 10 of 20

- ◯ Omit
- ◯ Possibly include
- ◯ Desirable
- ◯ Essential

Please provide the reason for your above response

Checklist item 11

OVERALL RESULTS OF THE STUDY

Harms

Depict the harms of the intervention according to adverse event data (e.g. post-surgical infection, pain) if possible using text and/or graphics (i.e. do not mention harms that were not assessed in the study)

Item 11 of 20

- ◯ Omit
- ◯ Possibly include
- ◯ Desirable
- ◯ Essential

Please provide the reason for your above response

Checklist item 12

STATISTICS

Point estimates and between-group differences

Present point estimates for between-group differences in study outcomes where possible (e.g. Odd Ratios, Mean Differences)

Item 12 of 20

- ○ Omit
- ○ Possibly include
- ○ Desirable
- ○ Essential

Please provide the reason for your above response

Checklist item 13

STATISTICS

Measures of precision

Present measures of precision for between-group differences in study outcomes (e.g. 95% Confidence Intervals)

Item 13 of 20

- Omit
- Possibly include
- Desirable
- Essential

Please provide the reason for your above response

Checklist item 14

STATISTICS

Present absolute effects for dichotomous outcomes

For dichotomous outcomes, express between-group differences and measures of precision using absolute effects rather than relative effects

Item 14 of 20

- ◯ Omit
- ◯ Possibly include
- ◯ Desirable
- ◯ Essential

Please provide the reason for your above response

Checklist item 15

STATISTICS

Clinical importance of effects

Depict the magnitude of effects (between-group differences) in relation to known thresholds for clinical importance if possible using text and/or graphics

Item 15 of 20

- ◯ Omit
- ◯ Possibly include
- ◯ Desirable
- ◯ Essential

Please provide the reason for your above response

Checklist item 16

STUDY LIMITATIONS

Risk of bias/study limitations

Depict at least one key study limitation using text and/or graphics

Item 16 of 20

- ◯ Omit
- ◯ Possibly include
- ◯ Desirable
- ◯ Essential

Please provide the reason for your above response

Checklist item 17

STUDY LIMITATIONS

Certainty of evidence (applicable to systematic reviews)

For infographics summarising systematic reviews, depict the certainty of evidence (e.g. using GRADE) using text and/or graphics

Item 17 of 20

- ○ Omit
- ○ Possibly include
- ○ Desirable
- ○ Essential

Please provide the reason for your above response

Checklist item 18

CONCLUSION/TAKE AWAY MESSAGE

Directness

Frame the conclusion or take away message around the correct population, intervention, comparator, and outcome (i.e. do not over generalise the findings of the study)

Item 18 of 20

- ○ Omit
- ○ Possibly include
- ○ Desirable
- ○ Essential

Please provide the reason for your above response

Checklist item 19

CONCLUSION/TAKE AWAY MESSAGE

Primary outcome

Frame the conclusion or take away message on the primary outcome (i.e. do not just focus on statistically significant results)

Item 19 of 20

- Omit
- Possibly include
- Desirable
- Essential

Please provide the reason for your above response

Checklist item 20

CONFLICTS OF INTEREST

Report conflicts of interest if any have been identified in the main text. If no conflicts of interest were reported in the main text, there is no need to mention conflict of interest in the infographic

Item 20 of 20

- ○ Omit
- ○ Possibly include
- ○ Desirable
- ○ Essential

Please provide the reason for your above response

Other items

Please use this space to suggest any checklist items not mentioned above that might be needed or to provide any other comments

Powered by Qualtrics

Supplementary File 3. Participant characteristics and Delphi results

Table 1. Participant characteristics

**Demographics (n=92)**		**Descriptive statistics**
**Female, n (%)**		47 (51.1%)
**Age (years), mean (SD)**		42.3 (12.7)
**Survey duration (minutes), median (IQR)**		20 (9 to 38)
**Education, n (%)**		
	University (postgraduate degree)	76 (82.6%)
	University (undergraduate degree)	16 (17.4%)
**Employment, n (%)**		
	Employed full-time	62 (67.4%)
	Employed part-time or casual	23 (25.0%)
	Student	7 (7.6%)
**Background, n (%)***		
	Health professional	64 (69.6%)
	Researcher	56 (60.9%)
	Methodologist	10 (10.9%)
	Journal Editor	8 (8.7%)
	Infographics designer	7 (7.6%)
	Statistician	6 (6.5%)
	Patient or member of the public	3 (3.3%)
	Policy maker	1 (1.1%)
	Other	7 (7.6%)
**Developed/designed an infographic, n (%)**		66 (71.7%)
**Infographics (n=66)**		**n (%)**
**How many have you developed/designed? n (%)**		
	1	8 (12.1%)
	2-5	30 (45.5%)
	6-20	17 (25.8%)
	21-50	7 (10.6%)
	>50	4 (6.1%)

**How many were published in a journal? n (%)**		
	0	31 (47.0%)
	1	8 (12.1%)
	2-5	15 (22.7%)
	6-20	8 (12.1%)
	21-50	2 (3.0%)
	>50	2 (3.0%)
**Developed/designed an infographic of comparative studies, n (%)**		38 (57.6%)
**Infographics of comparative studies (n=66)**		**n (%)**
**How many have you developed/designed? n (%)**		
	1	6 (15.8%)
	2-5	15 (39.5%)
	6-20	13 (34.2%)
	21-50	3 (7.9%)
	>50	1 (2.6%)

IQR: interquartile range; n: number of participants; SD: standard deviation

*participants could select multiple options so percentages do not add to 100%.

Table 2. Item ratings from the *Round 1 survey* and the Steering Group's decision on each item

Items	Essential	Desirable	Possibly include	Omit	Decision
**1) STUDY DESIGN**<br><br>Include the study design (e.g. pre-post cohort study, randomised controlled trial, systematic review). Can be included in the infographic's title or study title as a citation	60 (65.2%)	18 (19.6%)	14 (15.2%)	0 (0%)	Re-word and ask participants if they are happy with the new wording (Yes vs. No)
**2) STUDY CHARACTERISTICS**<br><br>**Population**<br><br>Depict the population/participants (e.g. older people with chronic low back pain) using text and/or graphics	81 (88.0%)	5 (5.4%)	6 (6.5%)	0 (0%)	Re-word and ask participants if they are happy with the new wording (Yes vs. No)
**3) STUDY CHARACTERISTICS**<br><br>**Population**<br><br>Include at least one important quantitative characteristic of the population/participants (e.g. mean age, mean symptom duration)	31 (33.7%)	36 (39.1%)	23 (25.0%)	2 (2.2%)	Re-word and ask participants if they are happy with the new wording (Yes vs. No)
**4) STUDY CHARACTERISTICS**<br><br>**Intervention**<br><br>Depict the intervention (e.g. acupuncture) using text and/or graphics	77 (83.7%)	8 (8.7%)	7 (7.6%)	0 (0%)	Re-word and ask participants if they are happy with the new wording (Yes vs. No)
**5) STUDY CHARACTERISTICS**<br><br>**Intervention**	35 (38.0%)	43 (46.7%)	11 (12.0%)	3 (3.3%)	Re-word and ask participants if they are happy with the new wording (Yes vs. No)

Include at least one important quantitative characteristic of the intervention (e.g. drug dose, intervention duration)					
**6) STUDY CHARACTERISTICS**<br><br>**Comparator**<br><br>Depict the comparator (e.g. no treatment) using text and/or graphics	64 (70.0%)	22 (23.9%)	6 (6.5%)	0 (0%)	Re-word and ask participants if they are happy with the new wording (Yes vs. No)
**7) STUDY CHARACTERISTICS**<br><br>**Comparator**<br><br>Include at least one important quantitative characteristic of the comparator (e.g. drug dose, intervention duration)	26 (28.3%)	39 (42.4%)	23 (25.0%)	4 (4.4%)	Re-word and ask participants if they are happy with the new wording (Yes vs. No)
**8) STUDY CHARACTERISTICS**<br><br>**Outcome**<br><br>Depict the outcome's construct (e.g. mortality, pain) using text and/or graphics and clearly label outcomes as primary or secondary	72 (78.3%)	11 (12.0%)	9 (9.8%)	0 (0%)	Re-word and ask participants if they are happy with the new wording (Yes vs. No)
**9) STUDY CHARACTERISTICS**<br><br>**Outcome**<br><br>Describe how the primary outcome was assessed, including the scale of the assessment tool (e.g. physical function as assessed by the SF-36, 0-100 scale)	24 (26.1%)	35 (38.0%)	30 (32.6%)	3 (3.3%)	Re-word and ask participates to re-rate (omit, possibly include, desirable vs. essential)
**10) OVERALL RESULTS OF THE STUDY**<br><br>**Benefits**	53 (57.6%)	29 (31.5%)	9 (9.8%)	1 (1.1%)	Re-word and ask participants if they are happy with the

Depict the benefits of the intervention according to the outcomes assessed (e.g. improves mortality, reduces disease reoccurrence) using text and/or graphics (i.e. do not mention benefits that were not assessed in the study)					new wording (Yes vs. No)
**11) OVERALL RESULTS OF THE STUDY**<br><br>**Harms**<br><br>Depict the harms of the intervention according to adverse event data (e.g. post-surgical infection, pain) if possible using text and/or graphics (i.e. do not mention harms that were not assessed in the study)	35 (38.0%)	34 (37.0%)	19 (20.7%)	4 (4.4%)	Re-word and ask participants if they are happy with the new wording (Yes vs. No)
**12) STATISTICS**<br><br>**Point estimates and between-group differences**<br><br>Present point estimates for between-group differences in study outcomes where possible (e.g. Odd Ratios, Mean Differences)	25 (27.2%)	34 (37.0%)	27 (29.4%)	6 (6.5%)	Re-word and ask participates to re-rate (omit, possibly include, desirable vs. essential)
**13) STATISTICS**<br><br>**Measures of precision**<br><br>Present measures of precision for between-group differences in study outcomes (e.g. 95% Confidence Intervals)	27 (29.4%)	31 (33.7%)	26 (28.3%)	8 (8.7%)	Re-word and ask participates to re-rate (omit, possibly include, desirable vs. essential)
**14) STATISTICS**<br><br>**Present absolute effects for dichotomous outcomes**<br><br>For dichotomous outcomes, express between-group differences and measures of precision using absolute effects rather than relative effects	18 (19.6%)	30 (32.6%)	34 (37.0%)	10 (10.9%)	Ask participants for confirmation this item should be excluded (yes vs. no)

**15) STATISTICS**<br><br>**Clinical importance of effects**<br><br>Depict the magnitude of effects (between-group differences) in relation to known thresholds for clinical importance if possible using text and/or graphics	30 (32.6%)	35 (38.0%)	21 (22.8%)	6 (6.5%)	Re-word and ask participants if they are happy with the new wording (Yes vs. No)
**16) STUDY LIMITATIONS**<br><br>**Risk of bias/study limitations**<br><br>Depict at least one key study limitation using text and/or graphics	13 (14.1%)	29 (31.5%)	33 (35.9%)	17 (18.5%)	Ask participants for confirmation this item should be excluded (yes vs. no)
**17) STUDY LIMITATIONS**<br><br>**Certainty of evidence (applicable to systematic reviews)**<br><br>For infographics summarising systematic reviews, depict the certainty of evidence (e.g. using GRADE) using text and/or graphics	23 (25.0%)	40 (43.5%)	21 (22.8%)	8 (8.7%)	Re-word and ask participants if they are happy with the new wording (Yes vs. No)
**18) CONCLUSION/TAKE AWAY MESSAGE**<br><br>**Directness**<br><br>Frame the conclusion or take away message around the correct population, intervention, comparator, and outcome (i.e. do not over generalise the findings of the study)	55 (59.8%)	23 (25.0%)	11 (12.0%)	3 (3.3%)	Re-word and ask participants if they are happy with the new wording (Yes vs. No)
**19) CONCLUSION/TAKE AWAY MESSAGE**<br><br>**Primary outcome**	50 (54.4%)	24 (26.1%)	14 (15.2%)	4 (4.4%)	Re-word and ask participants if they are happy with the new wording (Yes vs. No)

Frame the conclusion or take away message on the primary outcome (i.e. do not just focus on statistically significant results)					
**20) CONFLICTS OF INTEREST** Report conflicts of interest if any have been identified in the main text. If no conflicts of interest were reported in the main text, there is no need to mention conflict of interest in the infographic	12 (13.0%)	33 (35.9%)	20 (21.7%)	27 (29.4%)	Ask participants for confirmation this item should be excluded (yes vs. no)

Table 3. Item ratings from the *Round 2 survey* and the steering group's decision on each item

**Re-worded version of items that, in the *Round 1 survey,* almost reached consensus to be included**					
	**Essential**	**Desirable**	**Possibly include**	**Omit**	**Decision**
**STUDY CHARACTERISTICS**<br><br>**Outcome**<br><br>Present what tool was used to assess the primary outcome(s).<br><br>**Explanation and examples:** The infographic should clearly present what tool was used to assess the primary outcome(s), including the scale of the tool (e.g. physical function as assessed by the SF-36, 0-100 scale). In some cases, this information may not be applicable or relevant (e.g. there is no need to explain how mortality was assessed).	30 (44.1%)	19 (27.9%)	16 (23.5%)	3 (4.4%)	Include re-worded version in draft
**RESULTS**<br><br>**How much it helps by**<br><br>Present point estimates for between-group differences to demonstrate the effect (or lack thereof) of the intervention on the primary outcome(s).<br><br>**Explanation and examples:** The infographic should clearly present the size of the effect using point estimates for between-group differences (e.g. Risk Ratio, Mean Difference). Absolute effects are preferred over relative effects. Presenting this information for secondary outcomes is optional. Point estimates can be presented using lay language (e.g. 50% more likely than X, 1 point less pain than X on a 0-10 scale).	38 (55.9%)	23 (33.8%)	4 (5.8%)	3 (4.4%)	Include re-worded version in draft
**RESULTS**<br><br>**How certain we are**	33 (48.5%)	18 (26.5%)	15 (22.1%)	2 (2.9%)	Include re-worded version in draft

Present measures of precision for between-group differences to demonstrate the (un)certainty of the effect of the intervention on the primary outcome(s).<br><br>**Explanation and examples:** The infographic should clearly present measures of precision for between-group differences (e.g. 95% Confidence Intervals). Presenting this information for secondary outcomes is optional. Measures of precision can be presented using lay language (e.g. 20% more likely to 80% more likely than X, 5 points less pain to 15 points less pain compared to X on a 0-100 scale).					
**New items from suggestions in the *Round 1 survey***					
	**Essential**	**Desirable**	**Possibly include**	**Omit**	**Decision**
**AUTHOR INFORMATION**<br><br>List the authors and their affiliations.<br><br>**Explanation and examples:** The infographic should clearly list the study authors and their affiliations.	9 (13.2%)	24 (35.3%)	24 (35.3%)	11 (16.2%)	Exclude
**FUNDING**<br><br>List all funding sources.<br><br>**Explanation and examples:** The infographic should clearly list all funding sources, including funding received by the authors to conduct the study, fellowships held by the authors, and any other funding that may be perceived as creating a potential conflict of interest.	16 (23.9%)	17 (25.4%)	23 (34.3%)	11 (16.4%)	Exclude
**Re-worded version of items that reached consensus to include in the *Round 1 survey***					
	**Yes**	**No**			**Decision**

**STUDY DESIGN**<br><br>Present the study design.<br><br>**Explanation and examples:** The infographic should clearly present the study design of the study it is summarising (e.g. pre-post cohort study, randomised controlled trial, systematic review) so readers can understand the level of evidence of the findings being presented. The study design does not need to be repeated if it is mentioned in the title of the infographic or as part of the study citation in the infographic.	56 (82.4%)	12 (17.7%)		Include re-worded version in draft
**STUDY CHARACTERISTICS**<br><br>**Participants**<br><br>Present the population/participants.<br><br>**Explanation and examples:** The infographic should clearly present the population/participants included in the individual study or systematic review, the setting and/or country, and the sample size. For example, "120 older people with chronic low back pain presenting to an Australian public hospital". Infographics summarising randomised controlled trials should present the number of participants randomised. Infographics summarising systematic reviews should present the number of studies included and number of participants from these studies who were randomised. This allows readers to assess whether all randomised participants were included in the data analysis.	56 (82.4%)	12 (17.7%)		Include re-worded version in draft
**STUDY CHARACTERISTICS**<br><br>**Participants**<br><br>Present at least one important quantitative characteristic of the population/participants.	47 (69.1%)	21 (30.9%)		Include re-worded version in draft

**Explanation and examples:** The infographic should present at least one important quantitative characteristic of the population/participants (e.g. mean age, mean symptom duration), particularly if relevant to understanding the population/participants or interpreting the results. For example, the distinction between an acute vs. degenerative meniscal tear may be important when considering the effects of arthroscopic meniscectomy.				
**STUDY CHARACTERISTICS**<br><br>**Intervention**<br><br>Present the intervention.<br><br>**Explanation and examples:** The infographic should clearly present the intervention (e.g. acupuncture, lumbar discectomy) and who delivered the intervention (e.g. physiotherapist, orthopaedic surgeon).	56 (82.4%)	12 (17.7%)		Include re-worded version in draft
**STUDY CHARACTERISTICS**<br><br>**Intervention**<br><br>Present at least one important quantitative characteristic of the intervention.<br><br>**Explanation and examples:** The infographic should present at least one important quantitative characteristic of the intervention (e.g. drug dose, intervention duration), particularly if relevant to understanding the intervention or interpreting the results. For example, "20 vs. 4 physiotherapy sessions following anterior cruciate ligament surgery" highlights a key difference between the intervention and comparator.	56 (82.4%)	12 (17.7%)		Include re-worded version in draft
**STUDY CHARACTERISTICS**<br><br>**Comparator**	53 (77.9%)	15 (22.1%)		Include re-worded version in draft

Present the comparator.<br><br>**Explanation and examples:** If there is a comparator (e.g. placebo, other treatments), the infographic should clearly present it. The infographic should present who delivered the comparator and whether it was the same person who delivered the intervention. For example, “one physician administered the active drug and placebo”.			
**STUDY CHARACTERISTICS**<br><br>**Comparator**<br><br>Present at least one important quantitative characteristic of the comparator.<br><br>**Explanation and examples:** The infographic should present at least one important quantitative characteristic of the comparator (e.g. drug dose, intervention duration), particularly if relevant to understanding the comparator or interpreting the results. For example, “60mg vs. 30mg duloxetine per day” highlights a key difference between the intervention and comparator.	51 (75.0%)	17 (25.0%)	Include re-worded version in draft
**STUDY CHARACTERISTICS**<br><br>**Outcome**<br><br>Present the primary outcome(s).<br><br>**Explanation and examples:** The infographic should clearly present the primary outcome(s) (e.g. mortality, pain). Presenting secondary outcomes is optional. If presenting primary and secondary outcomes, clearly labelling outcomes as primary or secondary will reduce the risk of selective reporting.	58 (85.3%)	10 (14.7%)	Include re-worded version in draft
**RESULTS**	57 (83.8%)	11 (16.2%)	Include re-worded

**Whether it helps**<br><br>Present the effect (or lack thereof) of the intervention on the primary outcome(s).<br><br>**Explanation and examples:** The infographic should clearly present whether the intervention had an effect (or none) on the primary outcome(s) relative to the comparator. For example, "knee arthroplasty improved physical function vs. structured exercise alone". The number of participants analysed should be presented so readers can compare it to the number of participants randomised. Presenting the effects (or lack thereof) of the intervention on secondary outcomes is optional.			version in draft
**RESULTS**<br><br>**How important are the effects?**<br><br>**Explanation and examples:** Present the magnitude of effects (between-group differences) for the primary outcome(s) in relation to known thresholds for clinical importance. The infographic may highlight whether the effects of the intervention on the primary outcome(s) are clinically important if established thresholds exist. This information can be integrated into the presentation of results (e.g. dotted line on a graph). We acknowledge the concept of clinical importance is fraught with controversy due to measurement issues and because clinical importance depends on several factors (e.g. what an individual considers important, cost, complexity and inconvenience of the intervention).	52 (76.5%)	16 (23.5%)	Include re-worded version in draft
**STUDY LIMITATIONS**<br><br>**Certainty of evidence (applicable to systematic reviews)**<br><br>For infographics summarising systematic reviews, present the certainty of evidence. Infographics summarising systematic reviews should present the certainty of evidence if it was assessed in the original paper. For example,	58 (85.3%)	10 (14.7%)	Include re-worded version in draft

Grading of Recommendations, Assessment, Development and Evaluations (GRADE) allows effects to be categorized as high-, moderate-, low- or very low-certainty. Presenting these ratings will allow readers to understand how certain they can be of the findings presented in the infographic.				
**CONCLUSION/TAKE AWAY MESSAGE**<br><br>**Directness**<br><br>If including a conclusion or take away message, ensure it is appropriate to the study population, intervention, comparator, and outcome so findings are not over-generalised.<br>**Explanation and examples:** Infographics with a conclusion or take away message should ensure the message mentions the study population, intervention, comparator, and outcomes included in the original study. For example, "Exercise training for colorectal cancer survivors during chemotherapy reduces cancer-related fatigue compared to non-exercise training usual care". Being vague about these elements or broadening the message to include different study populations, interventions, comparators, or outcomes could mislead readers. For example, "Exercise training reduces fatigue in cancer survivors". A conclusion or take away message may not be necessary if other sections of the infographic present similar information.	54 (79.4%)	14 (20.6%)		Include re-worded version in draft
**CONCLUSION/TAKE AWAY MESSAGE**<br><br>**Primary outcome**<br><br>If including a conclusion or take away message, ensure it focuses on the primary outcome(s).<br><br>**Explanation and examples:** Infographics with a conclusion or take away message should ensure the message focuses on the primary outcome(s) to avoid selective reporting of statistically significant results. Reporting findings from	57 (83.8%)	11 (16.2%)		Include re-worded version in draft

secondary outcomes is optional. A conclusion/take away message may not be necessary if other sections of the infographic present similar information.			
**Items where there was clear consensus to exclude but we asked people if it should be re-included in the checklist**			
	**Yes**	**No**	**Decision**
**STATISTICS**			
**Present absolute effects for dichotomous outcomes**<br><br>For dichotomous outcomes, express between-group differences and measures of precision using absolute effects rather than relative effects	6 (8.8%)	62 (91.2%)	Exclude
**STUDY LIMITATIONS**			
**Risk of bias/study limitations**<br><br>Depict at least one key study limitation using text and/or graphics	17 (25.0%)	51 (75.0%)	Exclude
**CONFLICTS OF INTEREST**			
Report conflicts of interest if any have been identified in the main text. If no conflicts of interest were reported in the main text, there is no need to mention conflict of interest in the infographic	23 (33.8%)	45 (66.2%)	Exclude

Preference for one of three possible re-wordings of the item about harms		
	**Preferred**	**Decision**
**RESULTS**<br>**Whether it harms**<br>**1)** Present the frequency of adverse events in all groups and some examples of important adverse events. Present if a study did not report or measure adverse events.<br>**Explanation and examples:** The infographic should clearly present the frequency of adverse events in the intervention and control groups. Examples of important adverse events can be used to help the reader understand which adverse events are common (e.g. post-operative pain), serious (e.g. pulmonary embolism), or important for another reason. The infographic should highlight when a study did not report adverse events (despite measuring them) or when a study did not measure them. Adverse events should only be presented if they occurred in the study.	8 (11.8%)	Exclude
**2)** Present the frequency of serious adverse events in all groups and some examples of serious adverse events. Presenting the frequency of minor adverse events is optional. Present if a study did not report or measure adverse events.<br>**Explanation and examples:** The infographic should clearly present the frequency of serious adverse events (e.g. pulmonary embolism) in the intervention and control groups. The infographic should highlight when a study did not report serious adverse events (despite measuring them) or when a study did not measure them. Serious adverse events should only be reported if they occurred in the study.	29 (42.7%)	Include in draft

**3)** Present the frequency of minor and serious adverse events in all groups and some examples of minor and serious adverse events. Present if a study did not report or measure adverse events.<br><br>**Explanation and examples:** The infographic should clearly present the frequency of minor (e.g. post-operative pain) and serious adverse events (e.g. pulmonary embolism) in the intervention and control groups. The infographic should highlight when a study did not report minor or serious adverse events (despite measuring them) or when a study did not measure them. Minor and serious adverse events should only be reported if they occurred in the study	21 (30.9%)		Exclude
None of the above	10 (14.7%)		
**Language**	**Yes**	**No**	
Is the language of the checklist was appropriate for all people who may be interested in developing an infographic?	45 (69.2%)	20 (30.8%)	

Supplementary File 4. Round 2 survey

Welcome to the survey

Welcome to the health and medical infographics project!

Thank you for completing our first survey exploring what information people consider important to include in infographics. To refresh your memory about this study, we have provided some information about the study below.

What is the aim of this project?

Researchers at the University of Sydney are doing this project to develop a checklist of essential items to report in infographics that summarise the findings of comparative studies of health and medical interventions (e.g. randomised controlled trials, systematic reviews).

A checklist to facilitate clear, transparent, and sufficiently detailed infographics summarising comparative studies of health and medical interventions is needed to improve the accuracy with which research findings are communicated and avoid research findings being misinterpreted if consumers (e.g. health professionals, researchers) do not refer to the main paper.

To develop a checklist, we need to explore what information people consider important to include in infographics.

What does participation involve?

Participation involves completing two surveys between January 2022 and June 2023.

You have already completed the first survey. Thank you!

The second survey is ready to be completed.

This survey will take approximately 15-20 minutes. Our researchers take your privacy very seriously and all responses will be anonymous. You can also exit from the survey at any time.

A Participant Information Sheet is available here. You should review and retain this information sheet before proceeding. Please read it carefully before making up your mind about taking part. If you have any questions, please get in touch with one of the research team using the phone numbers or emails listed in the information sheet.

The University of Sydney Human Research Ethics Committee has approved this study (Protocol number: 2021/723).

The next screen will ask for your consent.

Who can participate?
You must be 18 years or older and be able to read and write English to take part in this study.

We are looking for everyone who completed the first survey to complete the second survey. This includes a range of people including statisticians and methodologists, individuals who produce infographics for journals (e.g. Informatics Editors), policy makers, editors of journals from various fields of medicine and health, authors who have published or developed infographics, and consumers (e.g. health professionals, members of the public).

Thank you for supporting this important research.

Participant Consent Form

In giving my consent I acknowledge that:

✓	I have read the Participant Information Statement and have been given the opportunity to discuss the study and my involvement in it with the researcher/s.
✓	The procedures required and time involved (including any inconvenience, risk, discomfort or side effect, and their implications) have been explained to me, and my questions about the project have been answered to my satisfaction.
✓	I understand that participation is voluntary. I am under no obligation to consent.
✓	I understand that I can withdraw from the study at any time, without providing a reason and without suffering any penalty. This will not affect my relationship with the researcher/s or university.
✓	I understand that my involvement is strictly confidential and no information about me will be used in any way that reveals my identity.
✓	I understand that data from this study may be used again for future research purposes, but that all data is strictly confidential and no information about me will be used in any way that reveals my identity.
✓	I would like the researchers to contact me to inform me about the results of the study.

- ○ Yes, I would be happy to go on and complete the survey
- ○ No, I would prefer not to complete the survey

I consent to the future use of any data I provide for research purposes. I understand that before the researchers can use any data I provide, they must seek additional ethics approval.

- ○ Yes
- ○ No

I would like the researchers to contact me to inform me about the results of the study

- ○ Yes
- ○ No

Welcome to the Round 2 survey!
This survey should not take more than 15-20 minutes.

You do not have to complete the survey in one sitting. If you use the same computer or device, you can return to the survey at any time.

The survey will remain open for 3 weeks.

In the Round 1 survey, you rated and commented on a list of potential items to include in a checklist for infographics that summarise the findings of comparative studies of health and medical interventions (e.g. pre-post cohort studies, randomised controlled trials, systematic reviews).

You rated whether each proposed item should be **omitted** or kept in the checklist (and whether it is considered **possible**, **desirable** or **essential**).

You were asked to consider the following information when doing so.

1. Reporting of the item should **FACILITATE** accurate interpretation of a study's findings;
2. The item is likely relevant to **ALL** infographics summarising the findings of comparative studies of health and medical interventions (e.g. pre-post cohort studies, randomised controlled trials, systematic reviews);
3. The set of items represent the **MINIMUM** that should be reported in all infographics summarising the findings of comparative studies of health and medical interventions (items are not too detailed for a 'minimum reporting guideline');
4. Adding items may **REDUCE** the clarity and visual appeal of the infographic

Based on feedback from the Round 1 survey, we have categorised checklist items into 3 groups:

i. Items where there was **clear consensus** to **include**
ii. Items where there was **almost consensus** to **include**
iii. Items where there was **clear consensus** to **exclude**

Before starting the Round 2 survey, we want you to further understand the context of this checklist and some general principles that apply to every checklist item.

Context

Our checklist is designed to improve the reporting of infographics summarising the findings of comparative studies of health and medical interventions, including retrospective observational studies, pre-post cohort studies, randomised controlled trials and systematic reviews.

- It does **not** apply to infographics summarising comparative studies using other designs (e.g. case studies, case series, cross-sectional observational studies).
- It does **not** apply to infographics summarising prognostic studies, diagnostic studies, and other types of research studies.

The scope of our checklist is limited to the content of an infographic. For guidance on design, consult a graphic designer or existing guidelines on this topic (e.g. THE 7 G.R.A.P.H.I.C. PRINCIPLES OF PUBLIC HEALTH INFOGRAPHIC DESIGN https://visualisinghealth.files.wordpress.com/2014/12/guidelines.pdf).

Guiding principles that apply to all checklist items

- These are guidelines and may not perfectly suit the needs of all infographics
- All infographics should include a way for readers to access the journal article (e.g. through a citation, DOI, URL, or QR code)
- Information requested from a checklist item may be presented using text and/or graphics
- Information requested from a checklist item may be presented as a footnote
- Information requested from a checklist item does not need to be duplicated in different sections of the infographic to satisfy the item (e.g. if the infographic presents the study population/participants in one section of the infographic, it does not need to present the study population/participants in another section)
- Each checklist item is accompanied by an ‘Explanation and example(s)’ section to help users implement the item
- Information requested from a checklist item should be presented in a way that the intended audience would understand

With this in mind, we want you to answer some questions about our checklist items.

First, we want you to consider the **items that almost reached consensus to include.** These items have been re-worded based on your feedback**.**

In the tables below, we present the original item (left-hand column) and re-worded item (right-hand column). Re-worded items now include an ‘explanation and example(s)’ section in dot points.

Please indicate whether each **re-worded item** should be **omitted** or kept in the checklist (and whether it is considered **possible**, **desirable** or **essential**). Please provide the reason for your response in the comments section.

Please note: Any of the items below that reach consensus at this stage, may be combined with other items that reached consensus in the Round One survey.

**Original item**	**Re-worded item including explanation and example(s)**
**STUDY CHARACTERISTICS**<br><br>**Outcome**<br><br>**Original item:** Describe how the primary outcome was assessed, including the scale of the assessment tool (e.g. physical function as assessed by the SF-36, 0-100 scale)	**STUDY CHARACTERISTICS**<br><br>**Outcome**<br><br>**Reworded item:** Present what tool was used to assess the primary outcome(s).<br>- The infographic should clearly present what tool was used to assess the primary outcome(s), including the scale of the tool (e.g. physical function as assessed by the SF-36, 0-100 scale). In some cases, this information may not be applicable or relevant (e.g. there is no need to explain how mortality was assessed).

- Omit
- Possibly include
- Desirable
- Essential

Please provide the reason for your above response

**Original item**	**Re-worded item including explanation and example(s)**
**Original item: STATISTICS**<br><br>**Point estimates and between-group differences**<br><br>Present point estimates for between-group differences in study outcomes where possible (e.g. Odd Ratios, Mean Differences)	**Re-worded item: RESULTS**<br><br>**How much it helps by**<br><br>Present point estimates for between-group differences to demonstrate the effect (or lack thereof) of the intervention on the primary outcome(s).<br>- The infographic should clearly present the size of the effect using point estimates for between-group differences (e.g. Risk Ratio, Mean Difference).<br>- Absolute effects are preferred over relative effects.<br>- Presenting this information for secondary outcomes is optional.<br>- Point estimates can be presented using lay language (e.g. 50% more likely than X, 1 point less pain than X on a 0-10 scale).

- Omit
- Possibly include
- Desirable
- Essential

Please provide the reason for your above response

**Original item**	**Re-worded item including explanation and example(s)**
**Original item: STATISTICS**<br><br>**Measures of precision**<br>Present measures of precision for between-group differences in study outcomes (e.g. 95% Confidence Intervals)	**Re-worded item: RESULTS**<br><br>**How certain we are**<br>Present measures of precision for between-group differences to demonstrate the (un)certainty of the effect of the intervention on the primary outcome(s).<br>- The infographic should clearly present measures of precision for between-group differences (e.g. 95% Confidence Intervals).<br>- Presenting this information for secondary outcomes is optional.<br>- Measures of precision can be presented using lay language (e.g. 20% more likely to 80% more likely than X, 5 points less pain to 15 points less pain compared to X on a 0-100 scale).

- Omit
- Possibly include
- Desirable
- Essential

Please provide the reason for your above response

Next, we want you to consider the **items where there was clear consensus to include** in the Round 1 survey. We have re-worded some of these items based on your feedback and want to get your opinion about whether the re-wording is an improvement on the original wording.

Please indicate whether you are happy with how each item has been re-worded. If you are not happy with how an item has been re-worded, please leave a comment explaining your opinion at the bottom of this table.

**Original item**	**Re-worded item including explanation and example(s)**
**STUDY DESIGN**<br><br>**Original item:** Include the study design (e.g. pre-post cohort study, randomised controlled trial, systematic review). Can be included in the infographic's title or study title as a citation.	**STUDY DESIGN**<br><br>**Reworded item:** Present the study design.<br>- The infographic should clearly present the study design of the study it is summarising (e.g. pre-post cohort study, randomised controlled trial, systematic review) so readers can understand the level of evidence of the findings being presented.<br>- The study design does not need to be repeated if it is mentioned in the title of the infographic or as part of the study citation in the infographic.

Are you happy with how this item has been re-worded?

- ○ Yes
- ○ No (please explain the reason for your opinion)

STUDY CHARACTERISTICS	STUDY CHARACTERISTICS
**Participants**<br><br>**Original item:** Depict the population/participants (e.g. older people with chronic low back pain) using text and/or graphics.	**Participants**<br><br>**Reworded item:** Present the population/participants.<br>- The infographic should clearly present the population/participants included in the individual study or systematic review, the setting and/or country, and the sample size.<br>- For example, "120 older people with chronic low back pain presenting to an Australian public hospital".<br>- Infographics summarising randomised controlled trials should present the number of participants randomised. Infographics summarising systematic reviews should present the number of studies included and number of participants from these studies who were randomised. This allows readers to assess whether all randomised participants were included in the data analysis.

Are you happy with how this item has been re-worded?

- ○ Yes
- ○ No (please explain the reason for your opinion)

**STUDY CHARACTERISTICS**<br><br>**Participants**<br><br>**Original item:** Include at least one important quantitative characteristic of the population/participants (e.g. mean age, mean symptom duration)	**STUDY CHARACTERISTICS**<br><br>**Participants**<br><br>**Reworded item:** Present at least one important quantitative characteristic of the population/participants.<br>- The infographic should present at least one important quantitative characteristic of the population/participants (e.g. mean age, mean symptom duration), particularly if relevant to understanding the population/participants or interpreting the results.<br>- For example, the distinction between an acute vs. degenerative meniscal tear may be important when considering the effects of arthroscopic meniscectomy.

Are you happy with how this item has been re-worded?

- Yes
- No (please explain the reason for your opinion)

**STUDY CHARACTERISTICS**<br><br>**Intervention**<br><br>**Original item:** Depict the intervention (e.g. acupuncture) using text and/or graphics	**STUDY CHARACTERISTICS**<br><br>**Intervention**<br><br>**Reworded item:** Present the intervention.<br>- The infographic should clearly present the intervention (e.g. acupuncture, lumbar discectomy) and who delivered the intervention (e.g. physiotherapist, orthopaedic surgeon).

Are you happy with how this item has been re-worded?

- ◯ Yes
- ◯ No (please explain the reason for your opinion)

STUDY CHARACTERISTICS<br><br>**Intervention**	STUDY CHARACTERISTICS<br><br>**Intervention**
**Original item:** Include at least one important quantitative characteristic of the intervention (e.g. drug dose, intervention duration)	**Reworded item:** Present at least one important quantitative characteristic of the intervention.<br>- The infographic should present at least one important quantitative characteristic of the intervention (e.g. drug dose, intervention duration), particularly if relevant to understanding the intervention or interpreting the results.<br>- For example, “20 vs. 4 physiotherapy sessions following anterior cruciate ligament surgery” highlights a key difference between the intervention and comparator.

Are you happy with how this item has been re-worded?

- ◯ Yes
- ◯ No (please explain the reason for your opinion)

**STUDY CHARACTERISTICS**<br><br>**Comparator**<br><br>**Original item:** Describe the comparator (e.g. no treatment) using text and/or graphics	**STUDY CHARACTERISTICS**<br><br>**Comparator**<br><br>**Reworded item:** Present the comparator.<br>- If there is a comparator (e.g. placebo, other treatments), the infographic should clearly present it.<br>- The infographic should present who delivered the comparator and whether it was the same person who delivered the intervention.<br>- For example, “one physician administered the active drug and placebo”.

Are you happy with how this item has been re-worded?

- Yes
- No (please explain the reason for your opinion)

**STUDY CHARACTERISTICS**<br><br>**Comparator**<br><br>**Original item:** Include at least one important quantitative characteristic of the comparator (e.g. drug dose, intervention duration)	**STUDY CHARACTERISTICS**<br><br>**Comparator**<br><br>**Reworded item:** Present at least one important quantitative characteristic of the comparator.<br>- The infographic should present at least one important quantitative characteristic of the comparator (e.g. drug dose, intervention duration), particularly if relevant to understanding the comparator or interpreting the results.<br>- For example, “60mg vs. 30mg duloxetine per day” highlights a key difference between the intervention and comparator.

Are you happy with how this item has been re-worded?

- ◯ Yes
- ◯ No (please explain the reason for your opinion)

STUDY CHARACTERISTICS<br><br>**Outcome**<br><br>**Original item:** Depict the outcome's construct (e.g. mortality, pain) using text and/or graphics and clearly label outcomes as primary or secondary	STUDY CHARACTERISTICS<br><br>**Outcome**<br><br>**Reworded item:** Present the primary outcome(s).<br>- The infographic should clearly present the primary outcome(s) (e.g. mortality, pain).<br>- Presenting secondary outcomes is optional.<br>- If presenting primary and secondary outcomes, clearly labelling outcomes as primary or secondary will reduce the risk of selective reporting.

Are you happy with how this item has been re-worded?

- ◯ Yes
- ◯ No (please explain the reason for your opinion)

Original item: OVERALL RESULTS OF THE STUDY	Re-worded item: RESULTS
**Benefits**<br><br>Describe the benefits of the intervention according to the outcomes assessed (e.g. improves mortality, reduces disease reoccurrence) using text and/or graphics (i.e. do not mention benefits that were not assessed in the study)	**Whether it helps**<br><br>Present the effect (or lack thereof) of the intervention on the primary outcome(s).<br>- The infographic should clearly present whether the intervention had an effect (or none) on the primary outcome(s) relative to the comparator.<br>- For example, “knee arthroplasty improved physical function vs. structured exercise alone”.<br>- The number of participants analysed should be presented so readers can compare it to the number of participants randomised.<br>- Presenting the effects (or lack thereof) of the intervention on secondary outcomes is optional.

Are you happy with how this item has been re-worded?

- ◯ Yes
- ◯ No (please explain the reason for your opinion)

Original item: STATISTICS	Re-worded item: RESULTS
**Clinical importance of effects**	**How important are the effects?**
Depict the magnitude of effects (between-group differences) in relation to known thresholds for clinical importance if possible using text and/or graphics	Present the magnitude of effects (between-group differences) for the primary outcome(s) in relation to known thresholds for clinical importance.<br>- The infographic may highlight whether the effects of the intervention on the primary outcome(s) are clinically important if established thresholds exist.<br>- This information can be integrated into the presentation of results (e.g. dotted line on a graph).<br>- We acknowledge the concept of clinical importance is fraught with controversy due to measurement issues and because clinical importance depends on several factors (e.g. what an individual considers important, cost, complexity and inconvenience of the intervention).

Are you happy with how this item has been re-worded?

- ◯ Yes
- ◯ No (please explain the reason for your opinion)

STUDY LIMITATIONS<br><br>**Certainty of evidence (applicable to systematic reviews)**<br><br>**Original item:** For infographics summarising systematic reviews, depict the certainty of evidence (e.g. using GRADE) using text and/or graphics	STUDY LIMITATIONS<br><br>**Certainty of evidence (applicable to systematic reviews)**<br><br>**Re-worded item:** For infographics summarising systematic reviews, present the certainty of evidence.<br>- Infographics summarising systematic reviews should present the certainty of evidence if it was assessed in the original paper.<br>- For example, Grading of Recommendations, Assessment, Development and Evaluations (GRADE) allows effects to be categorized as high-, moderate-, low- or very low-certainty.<br>- Presenting these ratings will allow readers to understand how certain they can be of the findings presented in the infographic.

Are you happy with how this item has been re-worded?

- ○ Yes
- ○ No (please explain the reason for your opinion)

CONCLUSION/TAKE AWAY MESSAGE	CONCLUSION/TAKE AWAY MESSAGE
**Directness**	**Directness**
**Original item:** Frame the conclusion or take away message around the correct population, intervention, comparator, and outcome (i.e. do not over generalise the findings of the study)	**Re-worded item:** If including a conclusion or take away message, ensure it is appropriate to the study population, intervention, comparator, and outcome so findings are not over-generalised.<br>- I fographics with a conclusion or take away message should ensure the message mentions the study population, intervention, comparator, and outcomes included in the original study.<br>- For example, "Exercise training for colorectal cancer survivors during chemotherapy reduces cancer-related fatigue compared to non-exercise training usual care".<br>- Being vague about these elements or broadening the message to include different study populations, interventions, comparators, or outcomes could mislead readers.<br>- For example, "Exercise training reduces fatigue in cancer survivors".<br>- A conclusion or take away message may not be necessary if other sections of the infographic present similar information.

Are you happy with how this item has been re-worded?

- ◯ Yes
- ◯ No (please explain the reason for your opinion)

CONCLUSION/TAKE AWAY MESSAGE	CONCLUSION/TAKE AWAY MESSAGE
**Primary outcome**	**Primary outcome**
**Original item:** Frame the conclusion or take away message on the primary outcome (i.e. do not just focus on statistically significant results)	**Re-worded item:** If including a conclusion or take away message, ensure it focuses on the primary outcome(s).<br>- I fographics with a conclusion or take away message should ensure the message focuses on the primary outcome(s) to avoid selective reporting of statistically significant results.<br>- Reporting findings from secondary outcomes is optional.<br>- A conclusion/take away message may not be necessary if other sections of the infographic present similar information.

Are you happy with how this item has been re-worded?

- Yes
- No (please explain the reason for your opinion)

For one checklist item that reached consensus to include, we would like your opinion on the best way to word it. Below is the original item, and then three reworded options which include an explanation and example. Green text highlights where passages of text have changed as compared to the first re-worded option.

Please tick the box next to the option you think is most appropriate.

If you don’t like any of them, we are open to other suggestions for re-wording.

Original item: OVERALL RESULTS OF THE STUDY

Harms

Depict the harms of the intervention according to adverse event data (e.g. post-surgical infection, pain) if possible using text and/or graphics (i.e. do not mention harms that were not assessed in the study)

Please select your preferred item from the options listed below:

○ **Re-worded item: RESULTS**

Whether it harms

Present the frequency of adverse events in all groups and some examples of important adverse events. Present if a study did not report or measure adverse events.

- The infographic should clearly present the frequency of adverse events in the intervention and control groups.
- Examples of important adverse events can be used to help the reader understand which adverse events are common (e.g. post-operative pain), serious (e.g. pulmonary embolism), or important for another reason.
- The infographic should highlight when a study did not report adverse events (despite measuring them) or when a study did not measure them.
- Adverse events should only be presented if they occurred in the study.

[illegible]

○ **Re-worded item: RESULTS**

Whether it harms

Present the frequency of serious adverse events in all groups and some examples of serious adverse events. Presenting the frequency of minor adverse events is optional. Present if a study did not report or measure adverse events.

- The infographic should clearly present the frequency of serious adverse events (e.g. pulmonary embolism) in the intervention and control groups.
- The infographic should highlight when a study did not report serious adverse events (despite measuring them) or when a study did not measure them.
- Serious adverse events should only be reported if they occurred in the study.

○ **Re-worded item: RESULTS**

Whether it harms

Present the frequency of minor and serious adverse events in all groups and some examples of minor and serious adverse events. Present if a study did not report or measure adverse events.

- The infographic should clearly present the frequency of minor (e.g. post-operative pain) and serious adverse events (e.g. pulmonary embolism) in the intervention and control groups.
- The infographic should highlight when a study did not report minor or serious adverse events (despite measuring them) or when a study did not measure them.
- Minor and serious adverse events should only be reported if they occurred in the study.

○ If you don’t like any of the options, please use this space to provide other suggestions for re-wording:

Next, we want you to consider the **items where there was clear consensus to exclude.** If you feel strongly about any of these items being included, please let us know which ones and your reason.

**Checklist item**
**STATISTICS**<br><br>**Present absolute effects for dichotomous outcomes**<br><br>For dichotomous outcomes, express between-group differences and measures of precision using absolute effects rather than relative effects
**STATISTICS**<br><br>**Present absolute effects for dichotomous outcomes**<br><br>For dichotomous outcomes, express between-group differences and measures of precision using absolute effects rather than relative effects
**STUDY LIMITATIONS**<br><br>**Risk of bias/study limitations**<br><br>Depict at least one key study limitation using text and/or graphics
**CONFLICTS OF INTEREST**<br><br>Report conflicts of interest if any have been identified in the main text. If no conflicts of interest were reported in the main text, there is no need to mention conflict of interest in the infographic

Do you think any of these items should be included?

- ◯ Yes
- ◯ No

If Yes, please specify which item you feel strongly about including and explain the reason for your opinion(s).

Next, we want to ask you about 2 new items we created based on your suggestions from the Round 1 survey.

Please indicate whether each proposed item should be **omitted** or kept in the checklist (and whether it is considered **possible**, **desirable** or **essential**). Please provide the reason for your response in the comments section.

AUTHOR INFORMATION
List the authors and their affiliations.

- The infographic should clearly list the study authors and their affiliations.

- ◯ Omit
- ◯ Possibly include
- ◯ Desirable
- ◯ Essential

Please provide the reason for your above response

FUNDING

List all funding sources.

- The infographic should clearly list all funding sources, including funding received by the authors to conduct the study, fellowships held by the authors, and any other funding that may be perceived as creating a potential conflict of interest.

- Omit
- Possibly include
- Desirable
- Essential

Please provide the reason for your above response

One final question…

Do you think the language of this checklist is appropriate for all people who may be interested in developing an infographic?

- Yes
- No (please explain the reason for your opinion)

Supplementary File 5. Detailed methods for the development of RIVA-C, findings and guide

1.1. Design and steering committee

We prospectively registered this reporting checklist on the Enhancing the QUAlity and Transparency Of health Research (EQUATOR) Network website[1] and developed it according to the Guidance for Developers of Health Research Reporting Guidelines.[2]

An international steering group led the development of RIVA-C. The steering group (led by JZ) consisted of information design experts (VE, WST, CW), individuals who produce infographics for journals (WST; Infographics Editor at The BMJ), individuals with experience in developing reporting guidelines (TH; led the development of the TIDieR checklist[3]), experts in clinical research methodology (CM, ME, IH, GF, JZ, MOK), editors of journals who publish infographics (ME, CA), authors who have published or developed infographics (JZ, GF, ME, IH), and health professionals (AG, IH).

1.2. Evidence from existing literature

Our review of 129 infographics summarising comparative studies of health and medical interventions identified potential checklist items with low adherence.[4] Items reported in fewer than half of infographics that could be feasible to incorporate included: potential harms of an intervention, measures of precision (e.g. 95% CIs), clinically important thresholds for effect sizes, risk of bias, certainty of evidence (for systematic reviews), study limitations, conclusions that considered risk of bias, and conflicts of interest. The steering group used these findings and other items from our analysis to develop a draft checklist for the first round of the Delphi survey (20 items) (Supplementary File 2).

1.3. Delphi survey

We performed a modified Delphi survey, with two rounds, to help decide on items that could potentially be included. We asked participants to consider the following guiding principles when reviewing items for inclusion:

1) Reporting of the item should facilitate accurate interpretation of a study's findings;
2) The item is likely relevant to all infographics summarising the findings of comparative studies of health and medical interventions (e.g. cohort studies, randomised controlled trials, systematic reviews);
3) The set of items represent the minimum that should be reported in all infographics summarising the findings of comparative studies of health and medical interventions (items are not too detailed for a 'minimum reporting guideline');

4) Adding items may reduce the clarity and visual appeal of the infographic

1.3.1. Round 1 survey methods

To maximise the generalisability and applicability of RIVA-C, we recruited individuals from the following participant groups to complete the *Round 1 survey*: statisticians and methodologists, individuals who produce infographics for journals (e.g. Infographic Editors), information design experts, policy makers, editors of journals from various fields of medicine and health, authors who have published or developed infographics, researchers, academics, health professionals, and patients or members of the public. Participants had to be 18 years of age or older and able to read and write English to participate.

Participants were invited to participate via Twitter and through snowballing. Members of the Steering Group also purposively suggested participants to approach via email and reached out to professional groups who might have members interested in participating (e.g. International Committee of Medical Journal Editors (ICMJE), International Society of Physiotherapy Journal Editors, International Institute for Information Design (IIID), Health Design Network, Design For All). Both the Twitter post and recruitment email included a link to complete the online survey in Qualtrics survey software (Qualtrics, Provo, UT, USA). The first page of the survey included a 'Welcome to the Study', the Participant Information Sheet, the Participant Consent Form, and a consent check box to proceed with the survey.

Participants answered questions about age, gender, educational attainment, employment status, professional background, and experience developing/designing infographics (Supplementary File 3). Participants were then asked to rate each proposed item of our draft checklist, with the following response options: omit, possibly include, desirable and essential. Participants were encouraged to provide rationale for their responses, to suggest alternative wording of proposed items, and to suggest additional items not listed in the survey. The *Round 1 survey* can be found in Supplementary File 2.

To analyse the data, we calculated frequencies of each response option for each item. We only analysed data from participants who rated every item. The views of all participants were given equal weight. For an item to reach consensus, the upper two response options (desirable or essential) needed to be rated by > 66% of participants. This threshold was based on previous studies that developed guidelines.[5, 6]

The Steering Group met via teleconference to discuss the findings of *the Round 1 survey* and refine the checklist for the *Round 2 survey.* Participant comments were used to refine the wording of items which reached or almost reached consensus, develop new items, and refine the scope of the checklist to better inform respondents to the *Round 2 survey.*

Email addresses were collected so participants could be contacted to complete the *Round 2 survey.* The survey remained open for three weeks, with a reminder email sent one week after the initial invitation and a final reminder sent after two weeks. Individuals who indicated that they wished to opt out of any subsequent surveys were not invited to complete the *Round 2 survey.*

1.3.2. Round 1 survey results

Of the 167 people who opened the survey, 141 consented to complete it (84%) and 92 (55%) provided complete responses and were included in the analysis. The mean age (standard deviation) of participants was 42 years (what was the SD), 51% were female, 83% had postgraduate education, and 67% were employed full time. Participants had various (and overlapping) professional backgrounds: health professionals (70%), researchers (61%), methodologists (11%), journal editors (9%), infographic designers (8%), statisticians (7%), patients or members of the public (3%), and policy makers (1%). Of the 72% who had developed an infographic before, 43% had developed six or more infographics. Further participant characteristics are in Supplementary File 3.

Participant ratings on checklist items and the Steering Group's decision for each item is in Supplementary File 3. Overall, there were 13 items which reached consensus and were re-worded for the *Round 2 survey* (to understand whether participants were happy with the new wording), three items which almost reached consensus and were re-worded for the *Round 2 survey* (so participants could re-rate the item), and three items which clearly did not reach consensus and were shown to participants in the *Round 2 survey* to see if any should be re-included in the checklist. In the *Round 1 survey,* there was clear consensus to include an item about reporting the potential harms of an intervention. However, since the steering group could not agree on the best way to word the item, we included three options in the *Round 2 survey* and asked participants to select which one they preferred.

1.3.3. Round 2 survey methods

Participants who rated every item in the *Round 1 survey* were invited to complete the *Round 2 survey* via email. Based on some participant comments in the *Round 1 survey,* we decided to further explain the context of the checklist and some general principles that apply to every checklist item in

the *Round 2 survey* (Box 1). We also added an 'explanation and examples' section to each re-worded or new potential checklist item.

Box 1. Further context and guiding principles provided to *Round 2 survey* participants

Context

Our checklist is designed to improve the reporting of infographics summarising the findings of comparative studies of health and medical interventions, including retrospective observational studies, pre-post cohort studies, randomised controlled trials and systematic reviews.

- It does **not** apply to infographics summarising comparative studies using other designs (e.g. case studies, case series, cross-sectional observational studies).
- It does **not** apply to infographics summarising prognostic studies, diagnostic studies, and other types of research studies.

The scope of our checklist is limited to the content of an infographic. For guidance on design, consult a graphic designer or existing guidelines on this topic (e.g. THE 7 G.R.A.P.H.I.C. PRINCIPLES OF PUBLIC HEALTH INFOGRAPHIC DESIGN https://visualisinghealth.files.wordpress.com/2014/12/guidelines.pdf).

Guiding principles that apply to all checklist items

- These are guidelines and may not perfectly suit the needs of all infographics
- All infographics should include a way for readers to access the journal article (e.g. through a citation, DOI, URL, or QR code)
- Information requested from a checklist item may be presented using text and/or graphics
- Information requested from a checklist item may be presented as a footnote
- Information requested from a checklist item does not need to be duplicated in different sections of the infographic to satisfy the item (e.g. if the infographic presents the study population/participants in one section, it does not need to present the study population/participants in another)
- Each checklist item is accompanied by an 'Explanation and example(s)' section to help users implement the item
- Information requested from a checklist item should be presented in a way that the intended audience would understand

Participants were then asked to:

i) Rate (omit, possibly include, desirable vs. essential) and provide comments on reworded versions of the three items that almost reached consensus to include and two new items we created based on suggestions from the *Round 1 survey*. An item was included in the draft checklist (to be discussed in the consensus meeting, see 2.3.5) if the upper two response options (desirable or essential) were rated by more than 66% of participants;[5, 6]

ii) State whether they were happy with re-worded versions of the 13 items where there was clear consensus to include (yes vs. no; people who responded 'no' were asked to provide a reason for their response). A re-worded version of an item was included in the draft checklist if > 50% of participants were happy with the revision;

iii) State whether any of the three items where there was clear consensus to exclude should be included again (yes vs. no; people who responded 'yes' were asked to provide a reason for their response). A previously excluded item was included in the draft checklist if > 50% wanted it to be re-included;

iv) Select their preference for one of three possible re-wordings of the item about harms; and

v) State whether the language of the checklist was appropriate for all people who may be interested in developing an infographic (yes vs. no; people who responded 'no' were asked to provide a reason for their response).

The *Round 2 survey* can be found in Supplementary File 4.

1.3.4. Round 2 survey results

There were 68 participants who completed the *Round 2 survey* (74% of respondents to the *Round 1 survey*). All three re-worded items that almost reached consensus in the *Round 1 survey* were included in the draft checklist (see 1.3.5). The two new items were excluded. All 13 re-worded items where there was clear consensus to include in the *Round 1 survey* were included in the draft checklist. None of the three items where there was clear consensus to exclude in the *Round 1 survey* were re-included in the checklist. The item about harms which focused on serious adverse events was the most popular option (43%) and 69% of participants said the language of the checklist was appropriate (Supplementary File 3).

1.3.5. Consensus meeting

We held an online consensus meeting with members of the steering group in February 2023 to discuss the results and feedback from the *Round 2 survey* and refine the draft checklist. Results of the *Round 2 survey* were sent to attendees prior to the meeting. Following the meeting, the project lead (JZ) refined the draft checklist and circulated it to the steering group for feedback. The checklist then underwent an iterative cycle of feedback from the steering group and revisions from the project lead (JZ) until the steering group was satisfied with the checklist and the examples used.

During the development of examples, the steering group realised it was important to achieve an appropriate balance between optimal reporting and practicality from a design perspective. This realisation led to the modification of several items. For example, the steering group and Delphi participants identified that it is important to include the number of studies included in a systematic review and number of participants from these studies who were randomised (overall and for each group). However, when we were developing examples with the BMJ infographic editor (WST), we realised this was not feasible for systematic reviews that had multiple interventions and comparisons. As a result, we added an 'if feasible' qualifier to this checklist item. We encountered a similar issue when reporting outcome values and the number of participants analysed across different groups and time points. To address this, we acknowledge in the checklist that it may not be feasible to include outcome values and number of participants analysed when multiple groups, outcomes or time points are presented.

1.4. Piloting and finalising RIVA-C

The draft version of RIVA-C was piloted by infographics editors or authors of infographics at The BMJ, Physiotherapy Evidence Database (PEDro – a research database of over 59,000 trials, systematic reviews and guidelines relevant to physiotherapy),[7] and Journal of Physiotherapy (#1 ranked journal in Rehabilitation and Orthopaedics) over a 6-month period. We asked for their feedback on RIVA-C, including whether the wording of any items or their explanation was ambiguous or difficult to interpret. Feedback from the piloting was summarised to the steering group via email where the members decided upon the final wording of the items, explanation, and examples.

RIVA-C was used by seven infographic developers and influenced the design of over 30 infographics. During piloting, the steering group realised it was important to achieve an

appropriate balance between optimal reporting and practicality from a design perspective. This realisation led to the modification of several items. For example, the steering group and Delphi participants initially identified that it was important to include the number of studies included in a systematic review and number of participants from these studies who were randomised (overall and for each group). However, during piloting, we realised this was not feasible for systematic reviews that had multiple interventions and comparisons. As a result, we added an 'if feasible' qualifier to this checklist item. We encountered a similar issue when reporting outcome values and the number of participants analysed across different groups and time points. To address this, we acknowledge in the checklist that it may not be feasible to include outcome values and number of participants analysed when multiple groups, outcomes or time points are presented.

References

1. Equator Network. Reporting guidelines under development for other study designs. https://www.equator-network.org/library/reporting-guidelines-under-development/reporting-guidelines-under-development-for-other-study-designs/#TERRI. Accessed Sept 11, 2023.

2. Moher D, Schulz KF, Simera I, et al. Guidance for Developers of Health Research Reporting Guidelines. *PLoS Med*. 2010;7(2):e1000217.

3. Hoffmann TC, Glasziou PP, Boutron I, et al. Better reporting of interventions: template for intervention description and replication (TIDieR) checklist and guide. *BMJ*. 2014;348:g1687.

4. Ferreira GE, Elkins MR, Jones C, et al. Reporting characteristics of journal infographics: a cross-sectional study. *BMC Med Educ*. 2022;22(1):326.

5. McInnes MDF, Moher D, Thombs BD, et al. Preferred Reporting Items for a Systematic Review and Meta-analysis of Diagnostic Test Accuracy Studies: The PRISMA-DTA Statement. *JAMA*. 2018;319(4):388-96.

6. Cohen JF, Korevaar DA, Gatsonis CA, et al. STARD for Abstracts: essential items for reporting diagnostic accuracy studies in journal or conference abstracts. *BMJ*. 2017;358:j3751.

7. PEDro - Physiotherapy Evidence Database. https://pedro.org.au/. Accessed July 29, 2023.

Supplementary File 6. The Reporting Infographics and Visual Abstracts of Comparative studies (RIVA-C) checklist

Section/item	Item No	Recommendation and explanation	Reported (Yes/No)
**Study characteristics**			
Study design	1	Present the study design.<br>• The infographic should clearly present the design of the study it is summarising (e.g., randomised controlled trial, systematic review, prospective cohort study).<br>• The study design does not need to be repeated if it is mentioned in the title of the infographic or as part of the study citation in the infographic.	
Population	2	Present the population/participants, sample size and important characteristics describing the population/participants<br>• The infographic should clearly present the population/participants and characteristics important to understanding the population/participants and interpreting the results (e.g., sample size, diagnosis, age, gender, socioeconomic status, symptom duration, study setting, country).<br>• Infographics summarising randomised controlled trials or non-randomised studies should present the number of participants randomised/enrolled (overall and for each group). Infographics summarising single-group studies should present the number of participants enrolled in the study. Infographics summarising systematic reviews should present the number of studies included and number of participants from these studies who were randomised/enrolled (overall and for each group, if feasible).	
Intervention and comparator	3	Present the intervention(s) and comparator(s) and important characteristics describing them.<br>• The infographic should clearly present the intervention(s) and comparator(s) (e.g., placebo, no treatment, other treatments). It should also present characteristics important to understanding the intervention(s) and comparator(s) and interpreting the results (e.g., drug type and dose, intervention duration, who delivered the intervention).<br>• Some studies will not have a comparator and only need to present the above information for the intervention.	
Outcomes	4	Present and clearly label the primary outcome(s), including the scale, units and time point(s).	

		• The infographic should clearly present the primary outcome(s) (e.g., mortality, pain), including the scale (e.g., 0 worst – 100 best), units (e.g., mmHg), and time point(s) of assessment, if relevant.<br>• Presenting secondary outcomes is optional.<br>• If presenting primary and secondary outcomes, clearly label which outcomes are primary to reduce the risk of selective reporting.<br>• If the study did not nominate a primary outcome, make this clear in the infographic (e.g., as a footnote).
**Results**		
How much it helps and how certain we are	5	Present between-group effects with measures of precision (e.g., mean difference and 95% CI) using absolute effects where possible, to demonstrate the effect (or lack thereof) of the intervention on the primary outcome(s) and the certainty of the effect.<br>• The infographic should clearly present the size (and certainty) of the effect on the primary outcome(s) using point estimates and measures of precision for between-group differences (e.g., Risk Difference or Mean Difference with 95% Confidence Intervals). Between-group differences are differences in outcomes between the intervention and control group(s) and are preferred to within-group changes (e.g., change from baseline to post-intervention). Within-group changes produce a biased effect of the intervention for several reasons (e.g., doesn't control for natural history of a disease, regression to the mean, etc.).<br>• When there isn't a comparator, the infographic should clearly present the size (and certainty) of the effect on the primary outcome using point estimates and measures of precision for within-group changes (e.g., Risk Difference or Mean Difference with 95% CI).<br>• The infographic should include the outcome values in each group (e.g., Mean of intervention vs. Mean of control) or at each time point where there isn't a comparator (e.g., Mean baseline vs. Mean post-intervention). However, we acknowledge this may not be feasible to include when multiple groups, outcomes or time points are presented.<br>• Absolute effects are preferred over relative effects (if available) because small absolute effects can appear large when expressed in relative terms (e.g., a decrease in risk from 1% to 0.5% equates to a 0.5% absolute decrease and 50% relative decrease). It is acceptable to present both absolute and relative effects.<br>• The number of participants analysed (or percentage drop out) in each group or at each time point should be presented so readers can compare it to the number of participants randomised or enrolled. This information may not be feasible

		to include when multiple groups, outcomes or time points are presented.<br>• Presenting point estimates and measures of precision for secondary outcomes is optional.<br>• Point estimates and measures of precision can be presented using lay language.
How important are the effects	6	When possible, present the magnitude of between-group effects for the primary outcome(s) in relation to justifiable thresholds for clinical importance.<br>• The infographic should highlight whether the between-group effects of the intervention on the primary outcome(s) are clinically important if justifiable thresholds exist. Justifiable thresholds are usually pre-specified by the authors (e.g. in the sample size calculation).<br>• This information can be integrated into the presentation of results (e.g. dotted line on a graph).
Whether it harms	7	Present the frequency of serious adverse events in each group and some examples of the most common serious adverse events if possible.<br>• The infographic should clearly present the frequency of serious adverse events in each group (e.g., serious adverse events: control = 10% vs. intervention = 5%), and some examples of the most common serious adverse events (e.g., pulmonary embolism: control = 5% vs. intervention = 2%).<br>• If a study does not report the overall frequency of serious adverse events in each group, adverse events can be reported in different ways (e.g., primary safety outcome in each group, all adverse events in each group, examples of common adverse events in each group or combined).<br>• Presenting the frequency of minor adverse events in each group and some examples of the most common minor adverse events is optional, unless it is important to understanding the safety of an intervention.<br>• The infographic should highlight when a study did not report adverse events (despite measuring them), when a study did not measure them, or when no serious adverse events occurred.
Certainty of evidence (applicable to systematic reviews)	8	Present the certainty of evidence for all effects presented in the infographic.<br>• For all outcomes for which effects are reported in the infographic, the certainty of evidence should be reported also (if certainty was assessed in the original paper). If certainty of evidence was not assessed in the original paper, make this clear in the infographic (e.g., as a footnote).<br>• Presenting the certainty of evidence will allow readers to

		understand how certain they can be of the findings presented in the infographic or whether more research is needed.
**Conclusion/take away message**		
Directness	9	When including a conclusion or take away message, ensure it is appropriate to the study population, intervention, comparator, and outcome.<br>• A conclusion or take away message that is appropriate to the study population, intervention, comparator, and outcomes will ensure findings are not over-generalised.<br>• A conclusion or take away message may not be necessary if other sections of the infographic present similar information.
Primary outcome	10	When including a conclusion or take away message, ensure it focuses on the primary outcome(s) and acknowledges potential harms of the intervention (as compared to the comparator).<br>• A conclusion or take away message that focuses on the primary outcome(s) will reduce selective reporting of statistically significant results. Acknowledging potential harms of the intervention, as compared to the comparator (if this data is available), will allow readers to weigh up efficacy and safety.<br>• Presenting findings from secondary outcomes is optional, with the exception of data on harms which is often a secondary outcome.<br>• A conclusion/take away message may not be necessary if other sections of the infographic present similar information.
